# Errors in protein synthesis increase the level of saturated fatty acids and affect the overall lipid profiles of yeast

**DOI:** 10.1371/journal.pone.0202402

**Published:** 2018-08-27

**Authors:** Ana Rita D. Araújo, Tânia Melo, Elisabete A. Maciel, Clara Pereira, Catarina M. Morais, Deolinda R. Santinha, Joana F. Tavares, Helena Oliveira, Amália S. Jurado, Vítor Costa, Pedro Domingues, Maria Rosário M. Domingues, Manuel A. S. Santos

**Affiliations:** 1 Department of Medical Sciences and Institute of Biomedicine–iBiMED, University of Aveiro, Aveiro, Portugal; 2 Mass Spectrometry Center, Department of Chemistry, QOPNA, University of Aveiro, Aveiro, Portugal; 3 Department of Biology, CESAM, University of Aveiro, Aveiro, Portugal; 4 i3S, Instituto de Investigação e Inovação em Saúde, Universidade do Porto, Porto, Portugal; 5 IBMC, Instituto de Biologia Molecular e Celular, Universidade do Porto, Porto, Portugal; 6 Departamento de Biologia Molecular, Instituto de Ciências Biomédicas Abel Salazar, Universidade do Porto, Porto, Portugal; 7 CNC, Center for Neuroscience and Cell Biology, University of Coimbra, Coimbra, Portugal; 8 Laboratory of Biotechnology and Cytomics, Department of Biology, CESAM, University of Aveiro, Aveiro, Portugal; 9 Department of Life Sciences, University of Coimbra, Coimbra, Portugal; Louisiana State University Health Sciences Center, UNITED STATES

## Abstract

The occurrence of protein synthesis errors (mistranslation) above the typical mean mistranslation level of 10^−4^ is mostly deleterious to yeast, zebrafish and mammal cells. Previous yeast studies have shown that mistranslation affects fitness and deregulates genes related to lipid metabolism, but there is no experimental proof that such errors alter yeast lipid profiles. We engineered yeast strains to misincorporate serine at alanine and glycine sites on a global scale and evaluated the putative effects on the lipidome. Lipids from whole cells were extracted and analysed by thin layer chromatography (TLC), liquid chromatography-mass spectrometry(LC-MS) and gas chromatography (GC). Oxidative damage, fatty acid desaturation and membrane fluidity changes were screened to identify putative alterations in lipid profiles in both logarithmic (fermentative) and post-diauxic shift (respiratory) phases. There were alterations in several lipid classes, namely lyso-phosphatidylcholine, phosphatidic acid, phosphatidylethanolamine, phosphatidylinositol, phosphatidylserine, and triglyceride, and in the fatty acid profiles, namely C16:1, C16:0, C18:1 and C18:0. Overall, the relative content of lipid species with saturated FA increased in detriment of those with unsaturated fatty acids. The expression of the *OLE1* mRNA was deregulated, but phospholipid fluidity changes were not observed. These data expand current knowledge of mistranslation biology and highlight its putative roles in human diseases.

## Introduction

Protein synthesis is highly accurate but, similarly to DNA replication and transcription, it is not an error free biological process [1]. Under typical physiological conditions both prokaryotic and eukaryotic cells mistranslate mRNAs with error rates that range from 10^−3^ to 10^−4^ [2], leading to the misincorporation of amino acids into proteins. These errors are readily dealt with by the mechanisms of protein quality control (e.g. molecular chaperones, proteasome and autophagy) [3,4] and normally do not cause significant damage to the cell. However, defects in the translational machinery that increase the level of error cause loss of fitness or even disease. For example, mutations in the human mitochondrial transfer RNA (tRNA) genes can lead to severe encephalomyopathies, such as myoclonic epilepsy and ragged-red fiber (MERRF) [5–8] and mitochondrial encephalomyopathy, lactic acidosis, and stroke-like episodes (MELAS) disease [8,9], among others [10,11] (Mitomap database). Alterations in genes that code for either mitochondrial or cytoplasmic aminoacyl-tRNA synthetases are also associated with disease [12,13]. Indeed, different mutations in the alanyl-RS gene can cause infantile mitochondrial cardiomyopathy [14], Charcot-Marie-Tooth disease type 2 [15], and the sticky syndrome in mice [16]. Faulty tRNA modification by the pseudouridine synthase Pus1p causes myopathy, lactic acidosis, and sideroblastic anemia (MLASA1) [17]. Mutations in ribosomal proteins have also been linked to anemia, and other pathologies [18].

Studies in yeast and bacteria showed that mistranslation produces antagonistic phenotypes [19–25]; in the cases where mistranslation is regulated it can be beneficial (for a review see [26]), but codon misreading in yeast decreases growth rate, alters cell morphology, increases reactive oxygen species (ROS) production, induces the petite phenotype, proteotoxic stress and gene expression deregulation, among other phenotypes [19–21,23,24,27–30]. The molecular nature of such phenotypic diversity is unknown.

Transcriptome analyses of yeast cells engineered to mistranslate at high level revealed that genes related to lipid metabolism were deregulated, e.g. fatty acid (FA) biosynthesis [21,24,29,31], but whether such deregulation alters lipid profiles is not yet clear. This is relevant to understand the mistranslation phenotype because lipids play many cellular roles beyond the classical role as structural membrane building blocks. They control membrane fluidity and permeability, participate in signaling cascades, e.g. apoptosis [32–34], and take part in the energetic metabolism, i.e. triacylglycerols (TG) and steryl esters [35]. They are important for correct protein function [36–41], protein-protein interaction [42–46], and act as proton sinks [47] in mitochondria. Importantly, cell membranes are remodeled in response to different stimuli [48], and constitutive perturbations in lipid homeostasis are relevant in the context of human pathologies, e.g. Stargardt-like macular degeneration [49–53] and Barth syndromes [54,55], among others [56].

To clarify the consequences of protein synthesis errors in lipid metabolism, yeast strains were constructed to express a recombinant Ser-tRNA that decodes Ala and Gly codons on a proteome wide scale [21]. The profiles of phospholipid (PL) and TG molecular species, and FA were then evaluated. Mistranslation induced significant alterations in several lipid species. We then investigated the occurrence of lipid peroxidation, assessed ROS levels, antioxidant enzyme activity, desaturation and membrane fluidity changes in an attempt to better understand our experimental observations.

## Materials and methods

### Strains and growth conditions

The *Saccharomyces cerevisiae* SY991 strain (EUROSCARF acc. no. Y40001; genotype: MATa *ura3*Δ0 *his3*Δ1 *leu2*Δ0 *trp1*Δ63 *ade2*Δ0 *lys2*Δ0 *ADE8*) was transformed with one of the following plasmids: pRS315 (control strain), pUA268 or pUA269 [21]. In brief, pUA plasmids are derived from pRS315 and contain a genomic DNA fragment of 150bp coding the *C*. *albicans* tRNA_TGA_^Ser^ gene, which anticodon was mutated to TGC (alanine) or TCC (glycine), corresponding to pUA268 and pUA269, respectively.

Transformations were performed using the LiAc/SS carrier DNA/PEG method [57] with minor alterations. Briefly, cells were grown overnight in YPD or selective medium, at 30°C and 180 rpm. A volume of 1 mL of cell suspension was pelleted at 8000 rpm and the supernatant was discarded. 1 to 3 μg of plasmid DNA were added to the transformation mix. After incubation and transformation mix removal, 200 μL of sterile water or selective medium were added to the transformed cells and plated onto appropriate selective media. Clones containing both plasmids were grown in minimal medium (0.67% yeast nitrogen base, 2% glucose, 0.2% drop-out mix). Plasmid inserts were verified by colony PCR and consecutive fragment sequencing.

Mistranslating strains were then characterized by determining growth rates as follows: cell cultures grown in 50 mL Erlenmeyer flasks were inoculated into new media to an initial OD_600_ = 0.02. Cell growth was monitored by measuring OD_600_ at several time points. Each growth rate corresponds to maximum growth of yeast cells in logarithmic phase and was calculated for each mistranslating strain by comparison to the control strain. Additionally, β-galactosidase protein activity and quantity, and tRNA expression were determined according to S1 and S2 Protocols.

### Protein isolation and quantification

Cell suspensions were spun down, washed twice and resuspended in phosphate-buffered saline (PBS) containing 0.2 mM phenylmethylsulfonyl fluoride (PMSF) to inhibit protease activity. For western blot analyses, other protease inhibitors (Complete, Mini, EDTA-free protease cocktail inhibitor tablets, Boehringer Mannheim) were also added. Cells were lysed with 0.5 mm glass beads in a homogenizer (Precellys). The extract was clarified by centrifugation at 4°C for 15 min at 13000 rpm. Protein concentration was determined by either the Bradford [58] or the BCA (Thermo scientific kit) methods, using bovine serum albumin (BSA) standards (Sigma).

### Detection of amino acid misincorporation

Tryptic digestion of protein samples was performed according to [59], with a few modifications. A protein band was manually excised from the gel and transferred to a microtube. The gel piece was washed once with 25 mM ammonium bicarbonate, three times with 25 mM ammonium bicarbonate in 50% acetonitrile (VWR Chemicals) and once with acetonitrile. Cysteine residues were reduced with 10 mM DTT and alkylated with 55 mM iodoacetamide. The gel piece was again washed as mentioned above, dried in a SpeedVac (Thermo Savant), and rehydrated in digestion buffer containing 12.5 μg/mL sequence grade modified porcine trypsin (Promega) in 50 mM ammonium bicarbonate. After 30 min, the supernatant was removed and discarded, 50 μL of 50 mM ammonium bicarbonate were added, and the sample was incubated overnight at 37°C. Extraction of tryptic peptides was performed by the addition of 5% formic acid (Fluka) once, followed by 5% formic acid and 50% ACN twice. Tryptic peptides were lyophilized in a SpeedVac (Thermo Savant) and resuspended in a 1% formic acid solution. The samples were analyzed with a QExactive Orbitrap (Thermo Fisher Scientific, Bremen) through the EASY-spray nano ESI source (Thermo Fisher Scientific, Bremen) coupled to an Ultimate 3000 (Dionex, Sunnyvale, CA) HPLC system. The trap column (100 μm I.D. x 2 cm packed with Acclaim PepMap RSLC C18, 5 μm 100 Å) and the EASY-spray analytical (75 μm I.D. x 75 cm packed with Acclaim PepMap RSLC C18, 2 μm 100 Å) columns were from Thermo Fisher Scientific. Peptides were trapped at 30 μl/min in 96% solvent A (deionized water with 0.1% formic acid). Elution was achieved with the solvent B (formic acid/ acetonitrile, 0.1:80 (V/V)) at 300 nl/min. The 92 min gradient used was as follows: 0–3 min, 96% solvent A; 3–70 min, 4–25% solvent B; 70–90 min, 25–40% solvent B; 90–92 min, 90% solvent B; 90–100 min, 90% solvent B; 101–120 min, 96% solvent A. The mass spectrometer was operated at 2 kV in the data-dependent acquisition mode. An MS2 method was used with an FT survey scan from 400 to 1600 m/z (resolution 70,000; AGC target 1E6). The ten most intense peaks were subjected to HCD fragmentation (resolution 17,500; AGC target 5E4, NCE 28%, max. injection time 100 ms, dynamic exclusion 35 s). Two subsequent LC-MS/MS runs were performed for each sample using exclusion lists containing all previously identified peptides. Spectra were processed and analyzed using Proteome Discoverer (version 2.2, Thermo), with MS Amanda and Sequest HT search engines. Uniprot (Swiss-Prot) protein sequence database (version of May 2016) was used for all searches under *Saccharomyces cerevisiae*. Database search parameters were as follows: carbamidomethylation of cysteine, alanine or glycine substitution by serine as a variable modification as well as oxidation of methionine, and the allowance for up to two missed tryptic cleavages. The peptide mass tolerance was 10 ppm, and fragment ion mass tolerance was 0.02 Da. To achieve a 1% false discovery rate, the Percolator node was implemented for a decoy database search strategy and peptides were filtered for high confidence and a minimum length of 6 amino acids, and proteins were filtered for a minimum number of peptide sequences of 1.

### Lipid extraction

Cells grown in a minimum of 200 mL of selective media were collected either in exponential (O.D._600_ 0.6–0.8) or PDS phase (O.D._600_ ~2.3). Cells were washed with PBS 1x and frozen in liquid nitrogen. The pellet was resuspended in 150 mM ammonium bicarbonate buffer and disrupted with 0.5 mm glass beads, by vortexing 1 min. Vortexing was repeated 8–10 times. The cell suspension was kept on ice between each vortexing cycle. Each extract was transferred to a new refrigerated Falcon tube, and pelleted at 8,000 rpm, for 5 min. Finally, the pellet was resuspended in 1 mL of ultra-pure distilled water. Total lipids from all yeast strains were extracted according to the Bligh and Dyer method [60]. Succinctly, 3.75 mL of chloroform/methanol 1:2 (V/V) were added to each sample, vortexed, and incubated on ice for a minimum of 30 min. Then, 1.25 mL of chloroform and 1.25 mL of ultra-pure distilled water were added, and the mixture was vortexed. Finally, samples were centrifuged at 1500 rpm for 5 min at room temperature to obtain a clear separation between the upper aqueous phase and the lower organic phase from which lipids were obtained. After transferring the organic phase to a clean tube, the extraction was repeated twice. The extracts were dried under a nitrogen flow, dissolved in chloroform, and stored at -20°C in amber glass vials.

### Phospholipid quantification

Quantification of the total amount of PL, as well as amounts of each PL class separated by TLC, was carried out with the phosphorus assay [61]. To quantify the total PL extract, 10 μL of each sample in duplicate were dried under a nitrogen flow, while quantification of the different classes separated by TLC, started by scraping the silica off the TLC plates directly to the quantification tubes. The quantity of phosphorus in the standards ranged from 0.1 to 2 μg. In brief, 650 μL of 65–70% perchloric acid were added to samples and standards, and the former were then incubated for 1 h at 200°C. Then, 3.3 mL of distilled water, 500 μL of a saturated ammonium molybdate solution, and 500 μL of a 10% (W/V) ascorbic acid solution were added sequentially to samples and standards, with intense vortexing in between additions. Finally, samples and standards were incubated for 5 min at 100°C in a water bath, and the absorbance was measured at 800 nm after cooling to room temperature. The samples containing silica particles obtained from TLC spots were spun down for 5 min at 4000 rpm before spectrophotometric determination. The relative abundance (%) of each PL class was calculated by relating the amount of PL in each spot to the total amount of PL in the sample.

### Separation of phospholipids classes by thin layer chromatography

PL classes from the total lipid extract were separated by thin layer chromatography (TLC) using silica gel plates with a concentrating zone 2.5x20 cm (Merck, Darmstadt, Germany). Prior to separation, plates were treated with 2.3% boric acid in ethanol. The plates with spots containing 30 μg of sample were developed using a solvent mixture consisting of chloroform/ethanol/water/triethylamine (30:35:7:35, V/V/V/V). Lipid spots on TLC plates were screened by exposure to primuline (50 μg/100 mL acetone/water, 80:20, V/V), under a UV lamp (λ = 254 nm). Identification of the different classes of PL was carried out by comparison to PL standards applied in the TLC plate. Then, each identified class spot was scraped and quantified by the phosphorus assay. For MS analysis, lipids were extracted from the TLC spots using chloroform/methanol (2:1, V/V).

### Analysis of fatty acid profiles by gas chromatography

Fatty acid (FA) profiling was evaluated by the analysis of fatty acid methyl esters (FAME), which were obtained by transesterification [62]. Briefly, 1 mL of hexane was added to 30 μg of dried phospholipid extract. FAME were obtained by sequentially adding 200 μL of 2 M KOH in methanol and 2 mL of saturated NaCl solution, followed by intense vortexing in between. After centrifugation, the organic phase was collected and dried under a nitrogen stream. The resulting FAME were dissolved in hexane prior to injection and analyzed on a PerkinElmer Clarus 400 gas chromatograph (Waltham, MA) equipped with a flame ionization detector (FID) and a DB-1 column with 30 m of length, 0.25 mm of internal diameter, and 0.1 μm of film thickness (J&W Scientific, Folsom, CA). The temperature of the injector was 250°C. The initial oven temperature was 50°C, the first ramp rate was 25°C/min to 180°C, held for 6min, followed by a second ramp rate of 40°C/min and the final temperature was 260°C, held for 3 min. The carrier gas was hydrogen, at a flow rate of 1.7 mL/min. Identification of FAME was done by comparison of retention times to those of a mixture of lipid standards (Sigma).

### Quantification of lipid hydroperoxides using the FOX II assay

Quantification of lipid hydroperoxides was achieved with the FOX II assay with minor alterations [63]. Aliquots of the total lipid extracts and standards were incubated with the FOX II reagent for 30 min in the dark. Hydrogen peroxide solutions were used as standards, with concentrations ranging from 0.01 to 0.4 mM. The solution was prepared as follows: 250 μM (NH_4_)_2_Fe(SO_4_).6H_2_O and 25 mM H_2_SO_4_ were dissolved in water, mixed with 4 mM 2,6-di-tert-butyl-p-hydroxytoluene (BHT), 100 μM xylenol orange and methanol. After incubation, the absorbance of samples and standards was read at 586 nm.

### Liquid chromatography—Mass spectrometry conditions for lipid analysis

For the LC-MS studies a hydrophilic interaction liquid chromatography (HILIC) was performed following mass spectrometry analysis. These HILIC-MS and HILIC-MS^n^ studies were conducted in a HPLC 2690 instrument (Waters Alliance, Milford, USA) equipped with an Ascentis Si column (15cm × 1mm, 3μm), and kept at room temperature. The HPLC was coupled to a linear ion trap (LXQ; Thermo Finnigan, San Jose, CA, USA) mass spectrometer. Two mobile phases were prepared as follows: mobile phase A (acetonitrile/methanol/H_2_O; 55:35:10 (V/V/V) with 10 mM ammonium acetate) and mobile phase B (acetonitrile/methanol 60:40 (V/V) with 10 mM ammonium acetate). Initially, 100% mobile phase B was held isocratically for 10 min, followed by a linear increase of mobile phase A to 100% within 10 min, which was maintained for 25 min. Finally, the mobile phase B was brought back to the initial elution conditions in 10 min. Aliquots of 25 μg of total phospholipid were diluted in 90 μL of mobile phase B, filtered, and 10 μL were injected into the column, using a flow rate of 40 μL/min.

The LXQ was operated in both positive (electrospray voltage +5 kV) and negative (electrospray voltage -4.7 kV) modes, at 275°C capillary temperature and a sheath gas flow of 8 U. Normalized collision energy (CE) was 27 (arbitrary units) for MS/MS. Data acquisition was carried out on an Xcalibur data system (V2.0). Relative quantification of individual phospholipid species was achieved by determination of the ratio between the area of reconstructed ion chromatogram of a given *m/z* value against the sum of the reconstructed areas considered for each class.

### Fractionation of lipid extracts by SPE

Total extracts were dissolved in n-hexane, chloroform and methanol (95:3:2) and fractionated by solid phase extraction (SPE) using Discovery DSC-NH_2_ SPE tubes (Supelco) and a Visiprep SPE vacuum manifold (Supelco). The SPE columns were activated with n-hexane and the neutral lipids were first eluted with chloroform, followed by elution of free fatty acids through the addition of diethyl ether and acetic acid [98:2(V/V)]. PL elution was then achieved by the addition of methanol and chloroform in different proportions [first, 6:1 (V/V) and then 1:1(V/V)]. The extracts were dried under nitrogen. The PL content was determined by the phosphorus method and this fraction was used for the fluorescence polarization study.

### Preparation of liposomes for fluorescence polarization

The lipid extracts dissolved in a chloroform/methanol [1:1(V/V)] mixture were evaporated to dryness under a stream of N_2_. The resulting film was hydrated with HBS (10 mM HEPES, 100 mM NaCl, pH 7.4) to obtain a phospholipid concentration of 100 μM. The lipid was dispersed at 55°C to form multilamellar vesicles.

### Incorporation of a fluidity probe and fluorescence polarization measurements

The fluidity probe 1,6-diphenyl-1,3,5-hexatriene (DPH) in dimethylformamide (DMF) was injected into liposome suspensions to obtain a lipid/probe molar ratio of 400/1 and then the mixtures were incubated at 55°C for 1 h in the dark. The fluorometric measurements were performed with a Perkin Elmer LS 55B fluorescence spectrophotometer (Perkin Elmer, U.S.A), equipped with polarization filters and a thermostated cell holder. The excitation was set at 336 nm and the emission at 450 nm (4 and 5 nm band pass). Light scattering from unlabeled liposome suspensions with equivalent volumes of DMF (blanks) had negligible effects on measurements. The degree of fluorescence polarization (P) was calculated according Shinitzky and Barenholz [64], from the equation P = (I_∥_-I_⊥_G)/(I_∥_+I_⊥_G), where I_∥_ and I_⊥_ are the intensities of the light emitted with its polarization plane parallel and perpendicular to the plane of the excitation light, respectively. G is the correction factor for the optical system, given by the ratio of vertically to the horizontally polarized emission components when the excitation light is polarized in the horizontal direction.

### Total RNA extraction

A volume of 50 mL of exponentially growing yeast cells or in early PDS phase were harvested by centrifugation, supernatants removed and the pellets were stored at -80°C. Total RNA was isolated using the acidic hot phenol method. Cell pellets were resuspended in acid phenol/chloroform [Sigma, 5:1 (V/V), pH 4.7] and TES-buffer (10 mM Tris pH 7.5, 10 mM EDTA, 0.5% SDS), vortexed and incubated at 65°C during 1 h, with vortexing every 10 min. Samples were centrifuged for 20 min at 13,000 rpm at 4°C and the water-phase was transferred to new microtubes containing phenol/chloroform [5:1 (V/V), pH 4.7]. The last step was repeated. Samples were vortexed and centrifuged for 10 min at 13,000 rpm at 4°C and the water-phase was transferred to new microtubes containing chloroform/isoamyl alcohol [Sigma, 25:1(V/V)]. Tubes were vortexed and centrifuged for 10 min at 13,000 rpm at 4°C. The water phase was transferred to new tubes containing 35 μL sodium acetate (3 M, pH 5.2) and 800 μL 100% ice cold ethanol. RNA was precipitated overnight at -30°C. After precipitation, tubes were centrifuged for 5 min at 13,000 rpm at room temperature. Then, the supernatant was removed, the pellets were washed with 80% ice cold ethanol, and centrifuged for 1 min at 13,000 rpm. Dried RNA pellets were resuspended in milliQ water and stored at -80°C.

### Reverse transcription

The RNA was first treated with DNAse I (amplification grade, Invitrogen), followed by phenol extraction. Briefly, deionized water and phenol/chloroform/isoamyl alcohol [25:24:1 (V/V)], at 4°C, were added to the treated samples and these were vortexed and centrifuged at 12,000 rpm, at 4°C, for 10 min. The water phase was transferred to a new microtube and chloroform was added. Again, the samples were vortexed and centrifuged. The water phase was transferred to new microtubes containing sodium acetate (3M, pH 5.2) and 100% ice cold ethanol and left at -80°C overnight. The pellet was washed with 80% ice cold ethanol and centrifuged at 8,000 rpm, at 4°C, for 10 min. The dried pellet was resuspended in deionized water. The synthesis of cDNA was accomplished by mixing 0.5 μg of RNA, 1 μL oligo dT_12-18_ primer (0.5 μg/μL, Invitrogen), 1 μL 10 mM dNTP mix (Thermo Scientific) and deionized water up to 12 μL. The mix was then incubated at 65°C, for 5 min, left 1 min on ice and spun down. Next, 1 μL of RNAse out, 2 μL 0.1 M DTT and 4 μL 5x first strand buffer were added and the mix was incubated at 42°C, for 2 min. Then, 1 μL SuperScript II reverse transcriptase (200 U/μL, Invitrogen) was added to each sample and incubated again for 50 min. Enzyme inactivation was achieved at 70°C, for 15 min. Finally, 1 μL of RNAse H (2500 U, Nzytech) was added and samples were incubated at 37°C, for 20 min. The negative controls were treated in the same way except that 1μL of deionized water was added instead of reverse transcriptase.

### RT-qPCR

Real time qPCR was performed in a 7500 system (Applied Biosystems). The reaction mix consisted of 10 μL of KAPA SYBR FAST Universal 2X qPCR Master Mix, 0.4 μL of each primer (10 pM final concentration), 6.8 μL of deionized H_2_O, and 2 μL of a 2-fold dilution of the cDNA. The primer sequences used were as follows: for *OLE1*, 5'-TGCCAATGTGGGACAAACAA (F), 5'-ACCACCTGGATGTTCAGAGA (R); for *ACT1*, 5’-AAGTGTGATGTCGATGTCCG (F), 5’-CCACCAATCCAGACGGAGTA (R). The thermocycling program consisted of a holding stage at 95°C for 3 min, followed by 40 cycles of 15 seconds at 95°C and 1 min at 60°C. The relative expression ratio was calculated according to the mathematical model established by Pfaffl [65]: R = [E_target_]^[ΔCt_target_(control-sample)] / [E_ref_]^[ΔCt_ref_(control-sample)].

### Quantification of total intracellular reactive oxygen species (ROS)

ROS levels were determined with dihydroethidium (DHE, Sigma) and dihydrorhodamine 123 (DHR123, Sigma), separately. For both dyes, 5x10^6^ cells were washed and resuspended in PBS. Then, A) DHE was added to a final concentration of 10 μg/mL and the cell suspension was incubated in the dark for 10 min, at 30°C. The cells were pelleted by centrifugation, washed twice with PBS and analyzed by flow cytometry (Coulter Epics XL, Beckman Coulter, Hialeah, FL, USA) with excitation/emission settings of 488/≥670nm (FL-3 channel), B) DHR123 was added to a final concentration of 15 μg/mL and the cell suspension was incubated in the dark for 1 h, at 30°C. The cells were pelleted by centrifugation, washed and resuspended in PBS and analyzed with excitation/emission settings of 488/525 nm (FL-1 channel). For all samples, a minimum of 10,000 events were counted. Data were acquired with the SYSTEM II software version 3.0 (Coulter Electronics, Hialeah, FL, USA) and analyzed with FlowJo software (Tree Star Inc., Ashland, OR-USA).

### Enzymatic activity

For all enzyme activities, yeast cells in post diauxic shift phase were harvested by centrifugation for 5 min at 4000 rpm. Cells were then resuspended in 36 mM potassium phosphate, pH 7.8 (SOD) or 50 mM sodium potassium phosphate, pH 7.0 (catalase) containing protease inhibitors (complete, mini, EDTA-free protease cocktail inhibitor tablets; Boehringer Mannheim). Total protein extracts were obtained by mechanical disruption through vortexing of the cell suspension in the presence of zirconium beads for 5 min. Short pulses of 30 s were applied followed by 30 sec incubation on ice. Cell debris was removed by centrifugation at 13000 rpm for 15 min and protein content was determined by the Lowry method, and bovine serum albumin used as a standard. To determine cytosolic and mitochondrial superoxide dismutase activity, 25 μg of total protein were loaded into 10% native PAGE gels, at 4°C. After electrophoresis, the gels were incubated in 2.5 mM nitro blue tetrazolium solution for 20 min, followed by incubation in a development solution (36 mM potassium phosphate, pH 7.8, 28 mM TEMED, 86 μM riboflavin) for 15 min. Finally, the gels were exposed to a 60 W light until the bands were visible. To determine cytosolic catalase activity, 80 μg of total protein were loaded into a 7.5% native PAGE gel, at 4°C. After electrophoresis, the gel was incubated in 2.5 mM horseradish peroxidase solution for 45 min, followed by addition of 5mM H_2_O_2_ and further incubated for 10 min. After washing, the gel was incubated with 50 μg/mL 3,3’-diaminobenzidine (Sigma) until the bands were visible. Band intensities were quantified by densitometry. Coomassie blue staining was used as loading control.

## Results

### Ser misincorporation at Ala and Gly sites

The occurrence of mistranslation was assessed in the two yeast strains carrying the heterologous Ser-tRNA expected to recognize the GCA Ala codon in one strain and the GGA Gly codon in the other. The presence of the tRNAs was verified by northern blot (S1 Fig) and the insertion of Ser at Ala and Gly sites was verified by mass spectrometry (MS) (Table 1, S1 and S3 Tables, S10 Fig). Additionally, the impact of mistranslation on the activity and quantity of the bacterial beta-galactosidase was assessed through the determination of enzymatic activity in the presence of its substrate and western blot, respectively (S2 Fig). There was reduction of beta-galactosidase activity in both mistranslating strains relative to the control, despite the unaltered protein quantity.

**Table 1:** Serine misincorporation counts identified by MS in peptides containing Ala and Gly sites. *codons decoded by the mutant tRNAs; NA, not applicable.

Ser misincorporation count				
		Control	Ser-tRNA^Ala^	Ser-tRNA^Gly^
**Ala**	GCA*	4	12	NA
	GCG*	0	1	NA
	GCT	49	42	NA
	GCC	23	19	NA
**Gly**	GGA*	1	NA	4
	GGG	1	NA	2
	GGT	26	NA	41
	GGC	3	NA	3

### Evaluation of alterations in the fatty acid profiles

The effects of mistranslation on the fatty acid (FA) profiles were assessed by analyzing fatty acid methyl esters by gas chromatography (GC). The relative content of FA determined for the two strains misincorporating Ser at Ala and Gly codon sites, and the control strain, was compared within each growth phase (logarithmic and post-diauxic shift, PDS). In total, 14 FA were identified in all three strains: C10:0, C12:0, C12:1, C14:0, C14:1, C16:0, C16:1, C18:0, 9c-C18:1, C18:1 (comparison to fatty acid methyl esters standards suggests a possible *trans* isomer), C20:0, C22:0, C24:0, C26:0. This composition in FA is typical of *S*. *cerevisiae* but FA profiles have been recognized to differ from strain to strain and also with different growth conditions [66,67]. Palmitoleic acid (C16:1) was the most abundant FA in all strains in both logarithmic and PDS phases. The second most abundant ones were palmitic acid (C16:0) in exponential phase and oleic acid (9c-C18:1) in PDS phase. All strains presented a lower amount of C18 FA in logarithmic phase than in PDS phase (Table 2).

**Table 2:** FA relative abundances are altered in the mistranslating strains.

**Logarithmic**						
	Control		Ser-tRNA^Ala^		Ser-tRNA^Gly^	
FA	mean	S.D.	mean	S.D.	mean	S.D.
C12:0	0.92	± 0.11	0.48	±0.01	0.62	±0.12
C14:1	0.51	± 0.05	0.43	±0.01	0.34	±0.01
C14:0	2.88	± 0.25	2.58	±0.04	2.02	±0.07
C16:1	48.42	± 0.27	45.52***	±0.27	46.67	±0.69
C16:0	21.91	± 0.55	23.55*	±0.08	23.70*	±0.62
C18:1n9c	20.42	± 0.70	21.62	±0.15	21.43	±0.06
C18:1	0.69	± 0.03	0.58	±0.04	0.51	±0.02
C18:0	4.25	± 0.38	5.23	±0.04	4.70	±0.04
∑SatFA	29.96	±0.58	31.85	±0.15	31.05	±0.63
∑MUFA	70.04		68.15		68.95	
**Post-Diauxic Shift**						
	Control		Ser-tRNA^Ala^		Ser-tRNA^Gly^	
FA	Av.	S.D.	Av.	S.D.	Av.	S.D.
C12:0	0.82	±0.09	0.66	±0.01	0.55	±0.10
C14:1	0.39	±0.02	0.36	±0.01	0.29	±0.02
C14:0	1.82	±0.02	1.90	±0.05	1.45	±0.11
C16:1	45.07	±0.30	40.31***	±0.25	41.08***	±0.41
C16:0	19.98	±0.49	21.76**	±0.29	21.78**	±0.79
C18:1n9c	24.88	±0.46	27.01***	±0.59	27.38***	±0.75
C18:1	1.28	±0.05	0.92	±0.06	0.93	±0.08
C18:0	5.76	±0.05	7.08*	±0.16	6.54	±0.27
∑SatFA	28.38	±0.53	31.40**	±0.44	30.32	±0.71
∑MUFA	71.62		68.60**		69.68	

FA profiles of control and mistranslating strains during logarithmic (log) and post-diauxic shift phases (PDS). Data are normalized to total peak area and presented as mean ± standard error (S.D.) of 3 biological replicates, except for log phase Ser-tRNA^Gly^ (2*n*). Two-way analysis of variance (ANOVA) followed by Bonferroni's multiple comparison test were performed (***P<0.001; **P<0.01; *P<0.05). (end of legend)

The FA profiles in logarithmic phase revealed differences between the control and the mistranslating strains. The relative amount of C16:1 decreased slightly but significantly in both engineered strains when compared to the control strain. Inversely, an increase in C16:0 relative abundance was observed. In PDS phase similar alterations were observed. Additionally, 9c-C18:1 was more abundant in both mistranslating strains in PDS phase and C18:0 relative content was higher exclusively in cells misincorporating Ser at Ala codons compared to the control strain (Table 2).

The comparison of the sums of saturated fatty acids (SFA) and mono unsaturated fatty acids (MUFA) showed a reduction trend of MUFA in both mistranslating strains that was statistically significant for the strain mistranslating Ser at Ala codons (Table 2). Overall, the induction of Ser misincorporation at Ala and Gly sites led to alterations in FA profiles, with higher saturation levels to which C16:0 and C18:0 FA contributed the most.

### Phospholipid and triacylglycerol profiles

**Fig 1:**
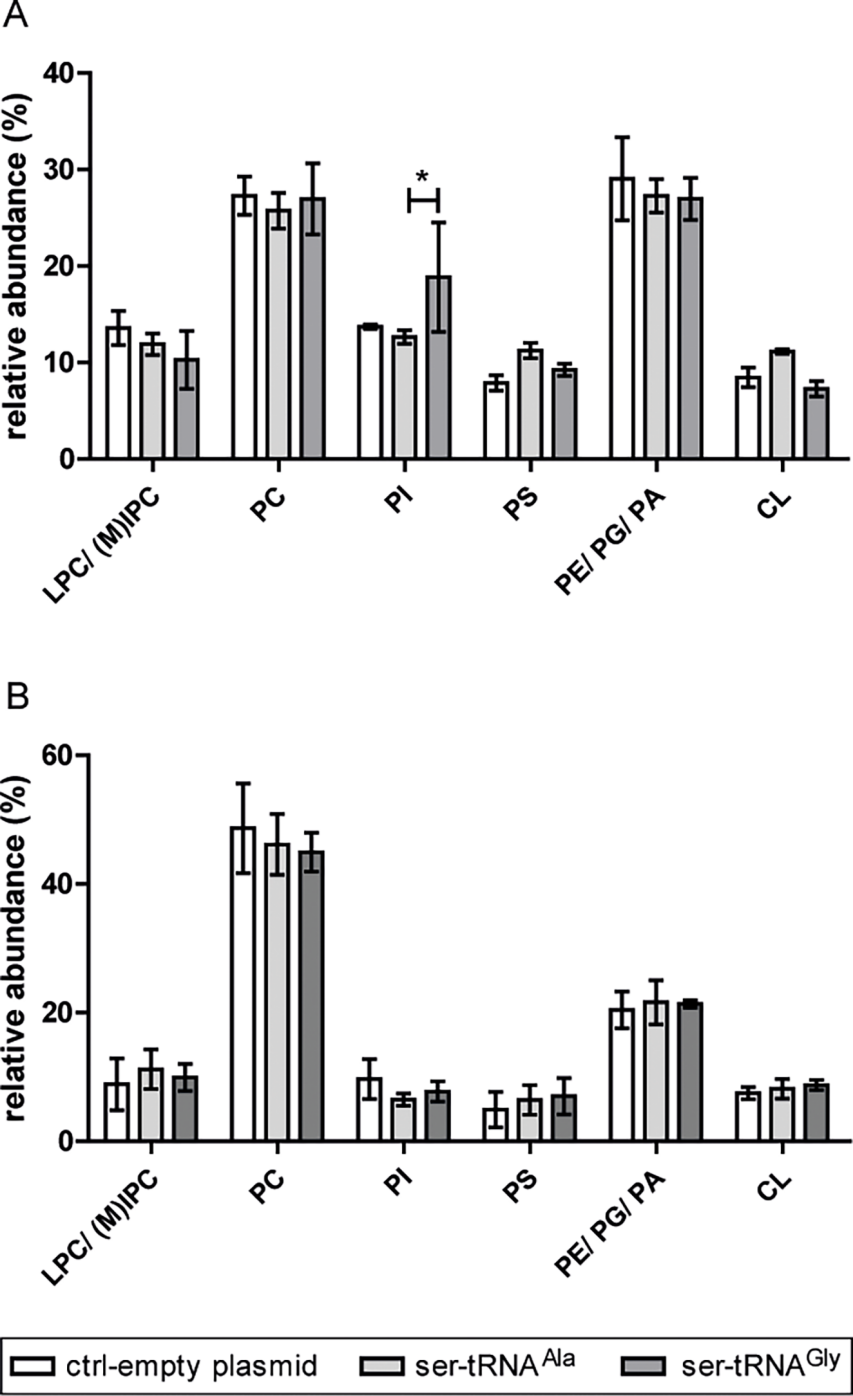
Phospholipid (PL) classes profiles are not affected in mistranslating strains. Relative abundance of phospholipid classes in logarithmic (A) and post-diauxic shift (B) phases. Phospholipids were fractionated by TLC and their identification was achieved by comparison with PL standards. MIPC and IPC were confirmed by direct infusion-ESI-MS^n^ analysis. Phospholipid classes were quantified by the phosphorus assay. Data were normalized against the sum of all values and are presented as mean ± standard deviation of three biological replicates. Statistical analysis was performed by two-way analysis of variance (ANOVA) followed by Bonferroni's multiple comparison test (*P<0.05).

To further evaluate how codon mistranslation affects the lipidome, phospholipid (PL) classes were separated by thin layer chromatography (TLC). Phospholipid identification was achieved by comparison with PL standards and the sphingolipids mannosyl-inositolphosphoceramides (MIPC) and inositolphosphoceramides (IPC) were further confirmed by electrospray (ESI)-MS^n^ analysis (S2 Table). PL classes were quantified by the phosphorus assay. The relative abundance of PL classes was determined for both growth phases (Fig 1) and the identified classes were the following: lyso-phosphatidylcholine (LPC), IPC and MIPC, phosphatidylcholine (PC), phosphatidylinositol (PI), phosphatidylserine (PS), phosphatidylethanolamine (PE), phosphatidic acid (PA) and cardiolipin (CL). All the classes were thoroughly identified in yeast elsewhere [68]. In log phase, the relative PL class content was the following: PC (26–27%); PA/ PE/ PG eluted together (27–29%); LPC/ IPC/ MIPC (10–14%); PI (13–19%); PS (8–11%); CL (7–11%). In PDS phase, the relative PL class content was the following: PC (45–49%); PA/ PE/ PG (20–22%); LPC/ IPC/ MIPC (9–11%); PI (6–10%); PS (5–7%); CL (8–9%). PC was the most abundant PL in both growth phases. In general, LPC/ IPC/ MIPC, PI, and PS classes showed higher abundance in log phase than in PDS phase.

The variation in CL and PS contents in log phase showed an increasing trend in the Ala-to-Ser relative to the control strain, but there were no statistical differences between control and mistranslating strains in any PL class, in neither growth phase. Among the recombinant strains, only a difference in PI was registered in logarithmic phase. Altogether these observations suggest that the bulk of PL classes profile was not significantly affected by the induction of mistranslation.

The LC-MS^n^ analysis allowed the identification and relative quantification, at the molecular level, of the profiles of the PL classes PC, PE, PG, PA, PI, PS and CL, as well as lyso-PL, namely LPC and LPE, and triacylglycerols (TG). The sphingolipids IPC and MIPC were also identified. The identified species are in accordance with previous studies [68–70]. A list of all the identified species can be found in S2 Table.

In PA, PE, PS and PC classes there were mainly five abundant species comprised of: 1) PL 32:2 (16:1/16:1), 2) PL 32:1 (16:1/16:0), 3) PL 34:2 (16:1/18:1), and 4&5) PL 34:1, assigned as both (18:1/16:0) and (18:0/16:1). In the PI class, the most abundant species had one mono unsaturated fatty acid (MUFA) (PI 32:1, PI 34:1, PI 36:1). The most abundant LPC species were LPC 16:1, 16:0, 18:1 and 18:0. Finally, the LPE and SP species showed low abundance and the signal to noise ratio did not allow the quantification of individual peaks.

**Fig 2:**
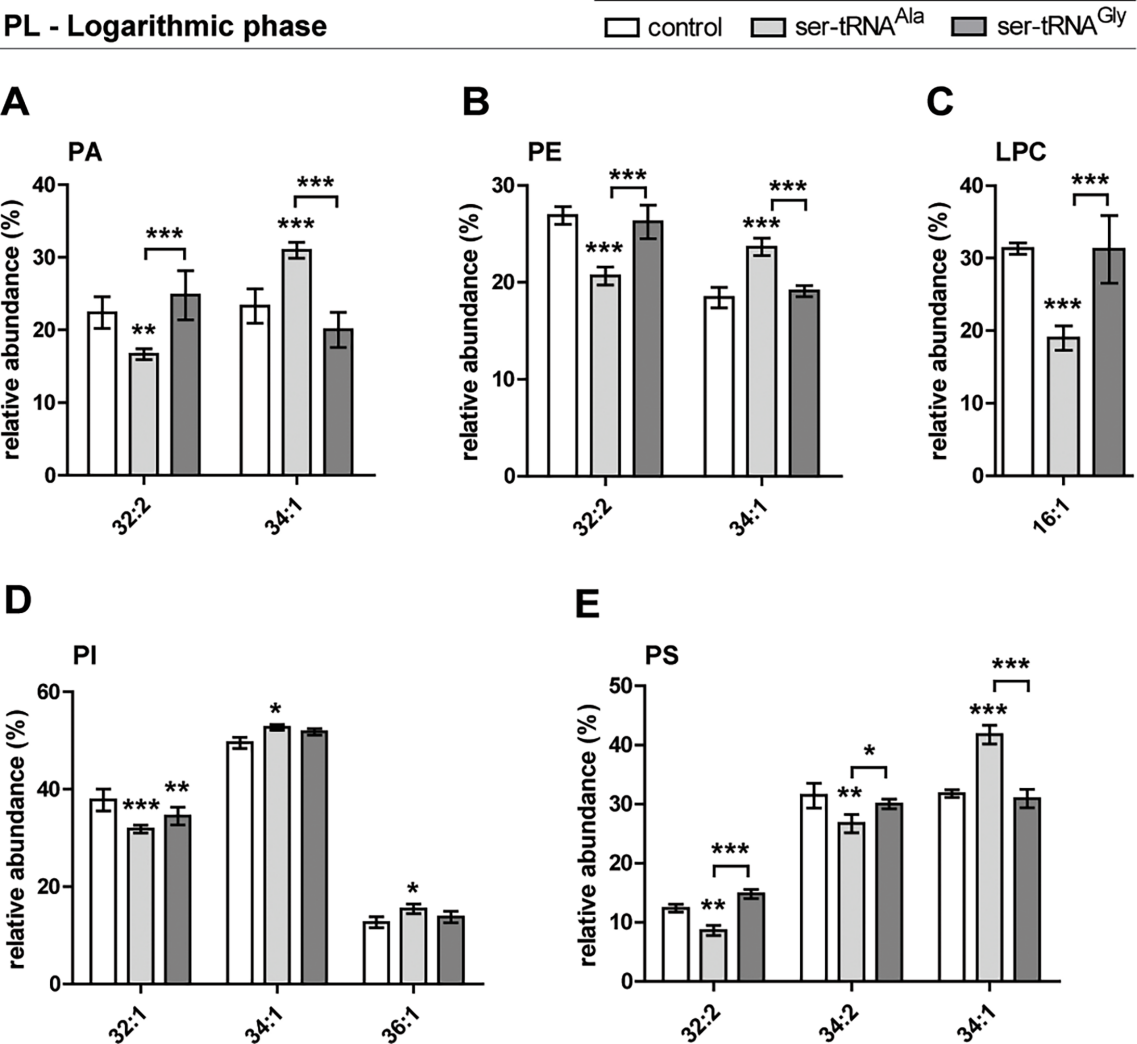
Differences in phospholipid (PL) molecular species profiles in logarithmic phase. Relative amounts of the molecular species that showed significant differences within each PL class–PA (A), PE (B), LPC (C), PI (D) and PS (E)–identified after comparison of the phospholipidomes from the mistranslating and control strains, and among each other, analyzed by HPLC-MS in negative mode (A, B, D, E) and positive mode (C. PL molecular species are identified as C:N (carbon number:number of double bonds). Data were normalized against the sum of all the reconstructed areas considered for each class and presented as mean ± standard deviation of three biological replicates. Statistical analysis was performed by two-way analysis of variance (ANOVA) followed by Bonferroni's multiple comparison test (***P<0.001 **P<0.01; *P<0.05). For the profile of all molecular species in each PL class see S3 Fig.

In log phase the alterations in the PL molecular species profiles, when compared to the control strain, were mostly observed in the Ala-to-Ser strain, namely in molecular species of PA, PE, LPC, PI and PS (Fig 2 and S3 Fig). This strain showed decreased relative content of PA (32:2), PE (32:2), LPC (16:1), PI (32:1), PS (32:2) and PS (34:2), and an increase of PA (34:1), PE (34:1), PI (34:1), PI (36:1), and PS (34:1) molecular species (Fig 2, Table 3). In the Gly-to-Ser strain only the PI profile was significantly disturbed when compared to the control strain, showing a reduction of the relative content of PI (32:1) and a trend similar to the Ala-to-Ser strain regarding PI (34:1 and 36:1) (Fig 2, Table 3). There were no significant alterations regarding PC (S3 Fig, Table 3) and PG (not shown) profiles in the mistranslating strains. There were significant differences between the mistranslating strains in eight molecular species: PA (32:1, 34:1), PE (32:2, 34:1), LPC (16:1) and PS (32:2, 34:2 and 34:1) (Fig 2).

**Table 3:** Summary of the changes observed in PL and TG molecular species in each mistranslating strain.

	Log		PDS	
	Ser-tRNA^Ala^	Ser-tRNA^Gly^	Ser-tRNA^Ala^	Ser-tRNA^Gly^
PA 32:2	↓	=	↓	=
PA 34:1	↑	=	=	=
PE 32:2	↓	=	=	=
PE 32:1	=	=	↓	↓
PE 34:1	↑	=	↑	=
PS 32:2	↓	=	=	=
PS 34:2	↓	=	=	=
PS 34:1	↑	=	=	=
PC 32:2	=	=	↓	↓
LPC 16:1	↓	=	↓	=
PI 32:1	↓	↓	=	=
PI 34:1	↑	=	=	=
PI 36:1	↑	=	=	=
TG 44:1	=	↑	=	↑
TG 46:2	=	↓	=	=
TG 48:3	↓	↓	↑	↓
TG 48:1	↑	↑	=	=
TG 50:3	↓	↓	=	↓
TG 50:1	↑	↑	=	↑
TG 52:3	=	↓	=	=

**Fig 3:**
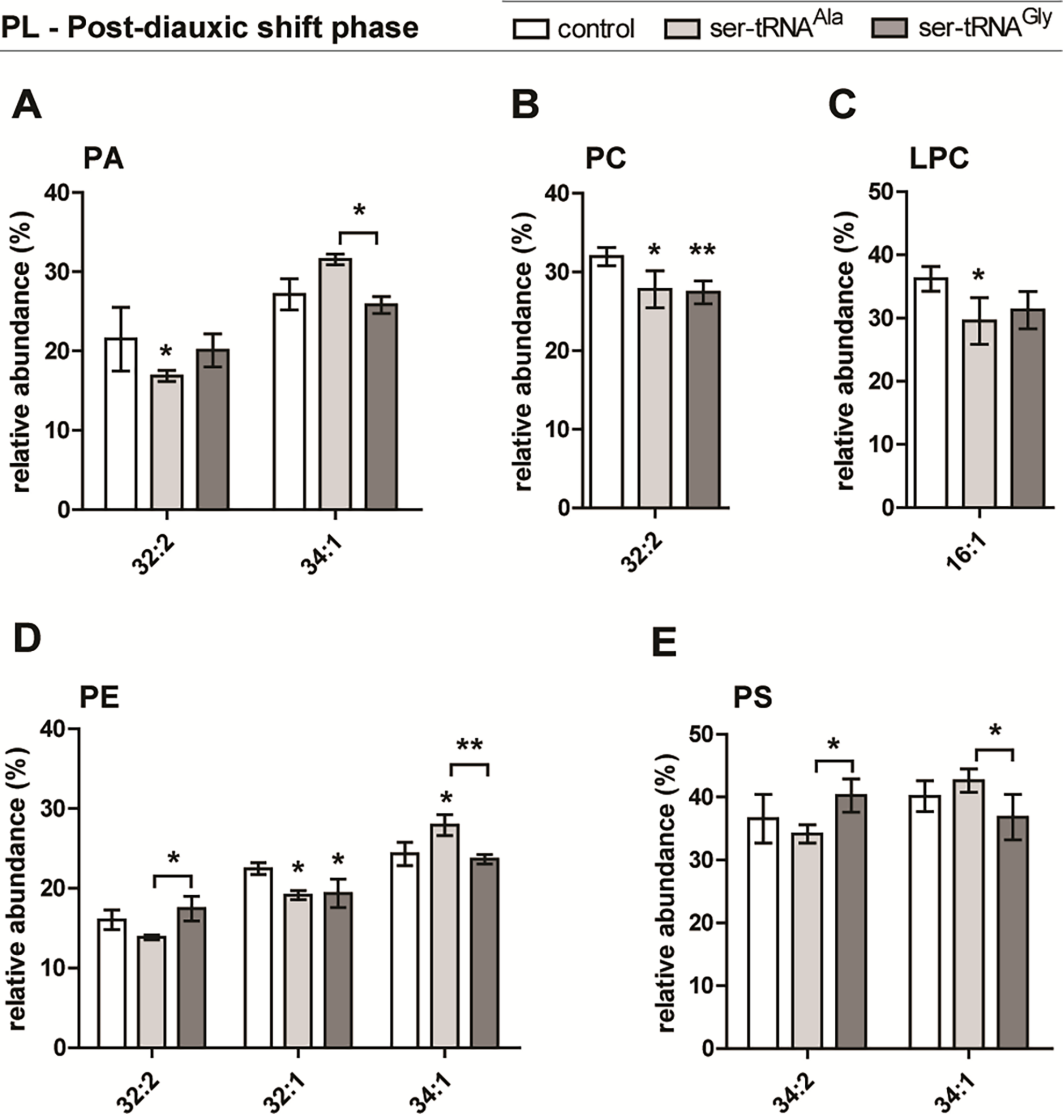
Differences in PL molecular species profile in PDS phase. Relative amounts of the molecular species that showed significant differences within each PL class–PA (A), PC (B), LPC (C), PE (D) and PS (E)–identified after comparison of phospholipidomes from the mistranslating and the control strains, and among each other, analyzed by HPLC-MS in negative mode (A, D, E) and positive mode (A, C). PL molecular species are identified as C:N (carbon number:number of double bonds). Data were normalized against the sum of all the reconstructed areas considered for each class and presented as mean ± standard deviation of three biological replicates. Statistical analysis was performed by two-way analysis of variance (ANOVA) followed by Bonferroni's multiple comparison test (***P<0.001 **P<0.01; *P<0.05). For the profile of all molecular species in each PL class see S4 Fig.

In the PDS phase the alterations in the PL molecular species profiles were again mostly observed in the Ala-to-Ser strain, relative to the control strain (Fig 3 and S4 Fig). The significantly altered profiles belong to PA, PC, LPC and PE classes. This strain showed a decreased relative content of PA (32:2), PC (32:2), LPC (16:0) and PE (32:1), and an increase of PE (34:1) molecular species only (Fig 3, Table 3). The Gly-to-Ser strain showed a significant reduction in the relative content of PC (32:2) and PE (32:1) (Fig 3, Table 3). The LPC profile variation was similar to that observed in the Ala-to-Ser strain though not statistically significant, and thus showed a reduction of the relative content of LPC (16:0). There were no significant alterations in the mistranslating strains regarding the PG (not shown), PI (S4 Fig) and PS profiles (Fig 3) relative to the control strain. Again, there were significant differences between the mistranslating strains regarding five molecular species PA (34:1), PE (32:2, 34:1) and PS (34:2, 34:1) (Fig 3).

Overall, for PA, PC and PS classes, the trend in the mistranslating strains relative to the control strain–mainly in the one misincorporating Ser at Ala sites–, was for lower relative content of species with 2 MUFA (e.g. PS 18:1/16:1), and higher relative content of species with 1 MUFA and 1 saturated FA (SFA) (e.g. PS 18:1/16:0 and PA 18:0/16:1) (Table 3). To some extent, such observations agree with the variations observed in the FA profiles. The same trend was seen for PE in exponential phase but not in PDS phase, where a decrease was observed for one of the species containing 1 MUFA and 1 SFA (Fig 3, Table 3).

**Fig 4:**
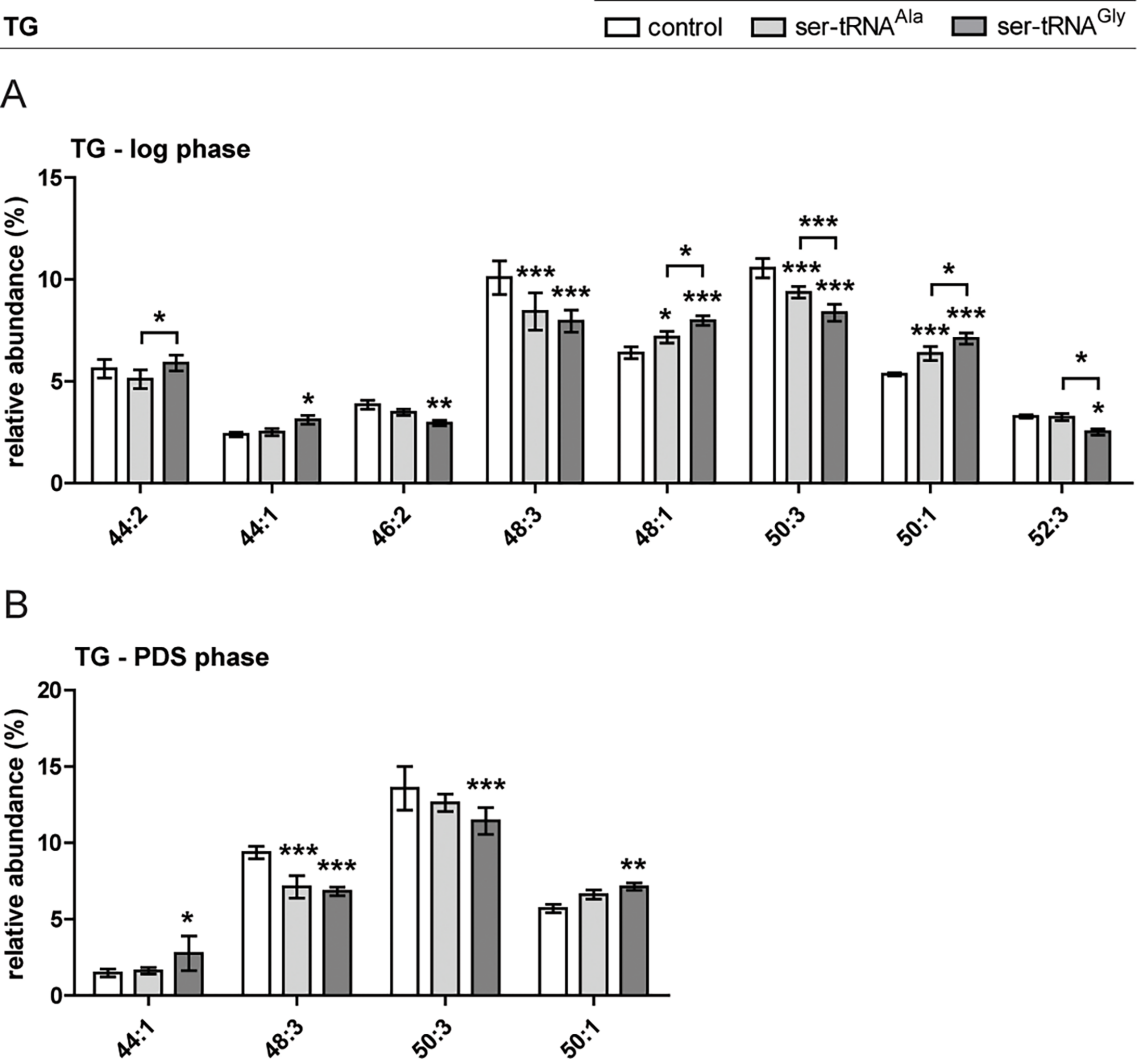
Differences in TG molecular species profile in logarithmic and PDS growth phases. Distribution of the relevant molecular species of TG in logarithmic (A) and post-diauxic shift phase (B) was altered. Data is normalized against the sum of the reconstructed areas considered for each phase and presented as mean ± standard deviation of three biological replicates. Statistical analysis was performed by two-way analysis of variance (ANOVA) followed by Bonferroni's multiple comparison test (***P<0.001 **P<0.01; *P<0.05). For the profile of all TG molecular species see S5 Fig.

The profiles of TG species in the total lipid extracts were also analyzed by LC-MS^n^. The most abundant TG species had either 3 C16 or 2 C16 and 1 C18 fatty acyl chains (Fig 4 and S5 Fig), as identified elsewhere [68–70]. Both in log and PDS phases, the profiles of the Gly-to-Ser strain varied the most relative to the control strain. The Ala-to-Ser strain showed log and PDS profiles variation similar to the other mistranslating strain but the differences were not always significant. The distribution of TG species in log phase (Fig 4A, Table 3) clearly showed a significant decrease of the relative content of TG (48:3) (mostly TG 16:1/16:1/16:1, but we also identified TG 14:1/16:1/18:1) and TG (50:3) (18:1/16:1/16:1), and an increase of TG (48:1) and (50:1) in the Ala-to-Ser strain relative to the control. In the Gly-to-Ser strain there was a decrease of TG (46:2), (48:3), (50:3) and (52:3) species, and an increase of TG (44:1), (48:1) and (50:1) species. In this strain, only one TG species bearing 2 unsaturated FA chains was significantly altered (46:2). The mistranslating strains were also statistically different from one another regarding the species TG (44:2), (48:1), (50:3), (50:1), and (52:3).

**Fig 5:**
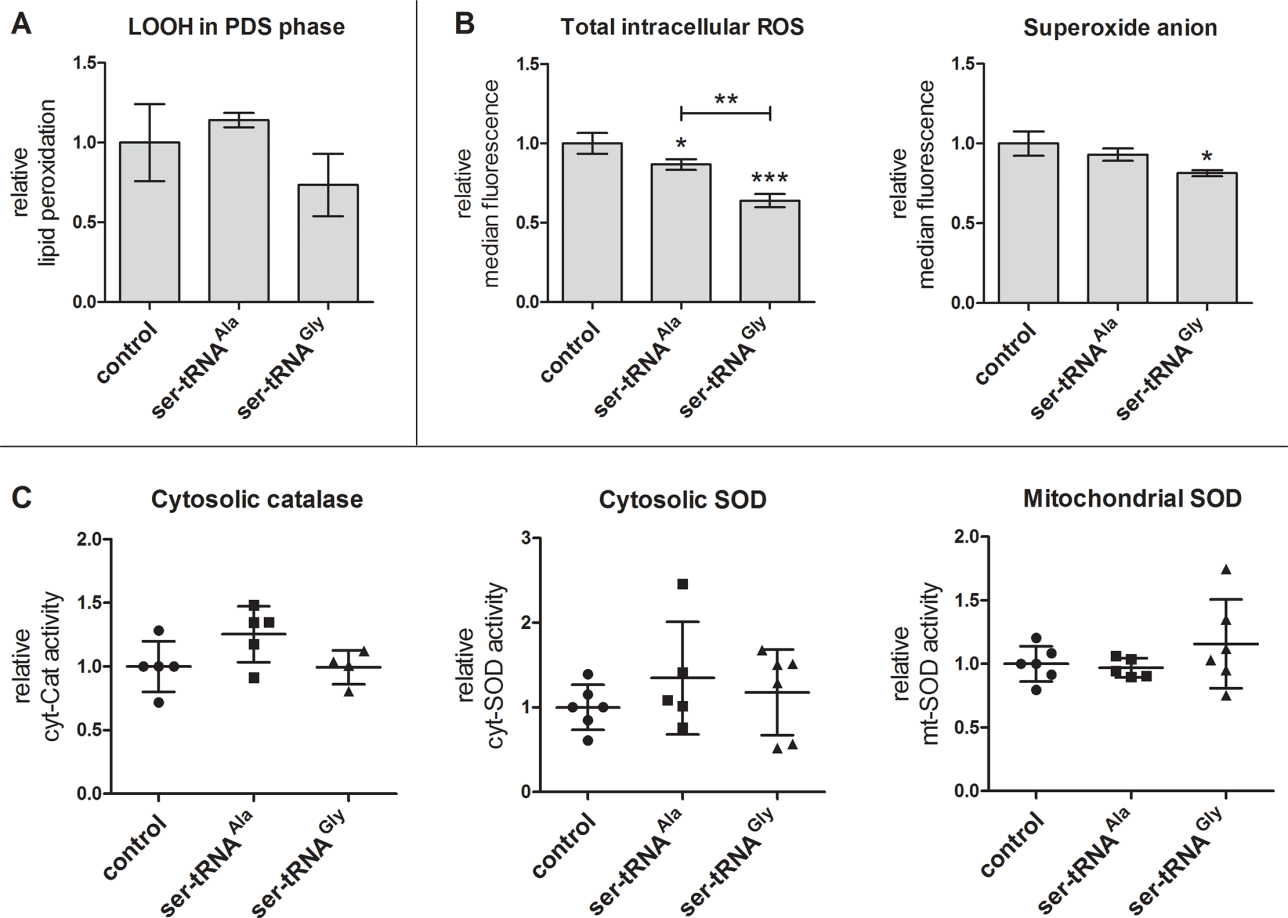
Assessment of oxidative stress markers in PDS phase. (A) Quantification of lipid hydroperoxides was achieved by performing the FOX II assay. Data were normalized against the averaged values of the control strain and are presented as mean ± standard deviation of duplicates of 3 biological replicates. (B) Total intracellular ROS and the superoxide anion alone were measured by flow cytometry using the DHR123 and DHE probes, respectively. Data are expressed as median values ± standard deviation of three biological replicates. (C) Enzymatic activities of cytosolic catalase and superoxide dismutase (SOD), and mitochondrial SOD were determined *in situ* after native-PAGE. Data are expressed as mean values ± standard deviation of three biological replicates assessed in two independent experiments. Statistical significance in all experiments was determined by one-way ANOVA, followed by Bonferroni’s multiple comparison test (*P<0.05; **P<0.01; ***P<0.001).

In PDS phase the lipid species distribution was similar, though a small shift towards the species with longer FA chains was observed (Fig 4 B and S5 Fig). Again, TG species with a total of 48 and 50 carbons were the most abundant, with TG (50:2) surpassing 15%. The alterations in the Ala-to-Ser strain were less evident, with only a significant decrease (~2%) in the relative content of TG (48:3) species. Regarding the Gly-to-Ser strain, TG species (48:3) and (50:3) were decreased while TG (44:1) and (50:1) showed an increment (Fig 5B). These results can also relate to the FA profile of Gly-to-Ser strain. Overall, there was an increase in the percentage of TG with 2 SFA and 1 MUFA and a decrease in TG with 3 MUFA.

### Oxidative damage is not responsible for the lipidome alterations in the mistranslating strains

Since amino acid misincorporation was previously shown to lead to oxidative damage (e.g. ROS, protein oxidation) [21,30,71,72], the decreased MUFA relative content in the FA and PL species profiles in the mistranslating strains led us to hypothesize that such observations could be due to FA oxidation. In pursuit for clarification we analyzed the levels of lipid hydroperoxides, which are the primary products of lipid oxidation. We observed the same variation trend in logarithmic (S6 Fig) and PDS growth phases (Fig 5A): the strain misincorporating Ser at Ala codons had a slightly higher level of lipid hydroperoxides, when compared to the control strain, while the Gly-to-Ser mistranslating strain showed a lower level. However, there were no statistically significant differences when compared to the control strain.

Earlier observations made in strains mistranslating Ala and Gly codons [21] led to the hypothesis that total ROS levels would be mostly unchanged in both strains. Additionally, an increase of the superoxide anion alone could be observed in a strain mistranslating Gly codons, possibly accompanied by an intensification in antioxidant defense activity (transcriptomic data). Thus, we quantified total ROS and the superoxide anion levels in the respiratory phase, in which ROS production was expected to be higher due to oxidative phosphorylation. We also verified whether the activities of the antioxidant enzymes catalase (cytosolic) and superoxide dismutase (SOD; cytosolic and mitochondrial isoforms) were elevated. Total ROS levels were decreased in both strains (Fig 5B). Surprisingly, decreased levels of the superoxide anion were also observed in the Gly-to-Ser strain, while no significant changes were seen for the other recombinant strain (Fig 5B). Additionally, the activities of all three antioxidant enzymes tested were unchanged when compared to the control (Fig 5C). These results suggest that these strains are not subjected to damaging oxidation levels. Instead, mistranslation likely caused an adjustment in lipid metabolism as suggested by the above mentioned transcriptomics studies [21,24,29,31].

### *OLE1* transcripts are altered in Gly-to-Ser background

**Fig 6:**
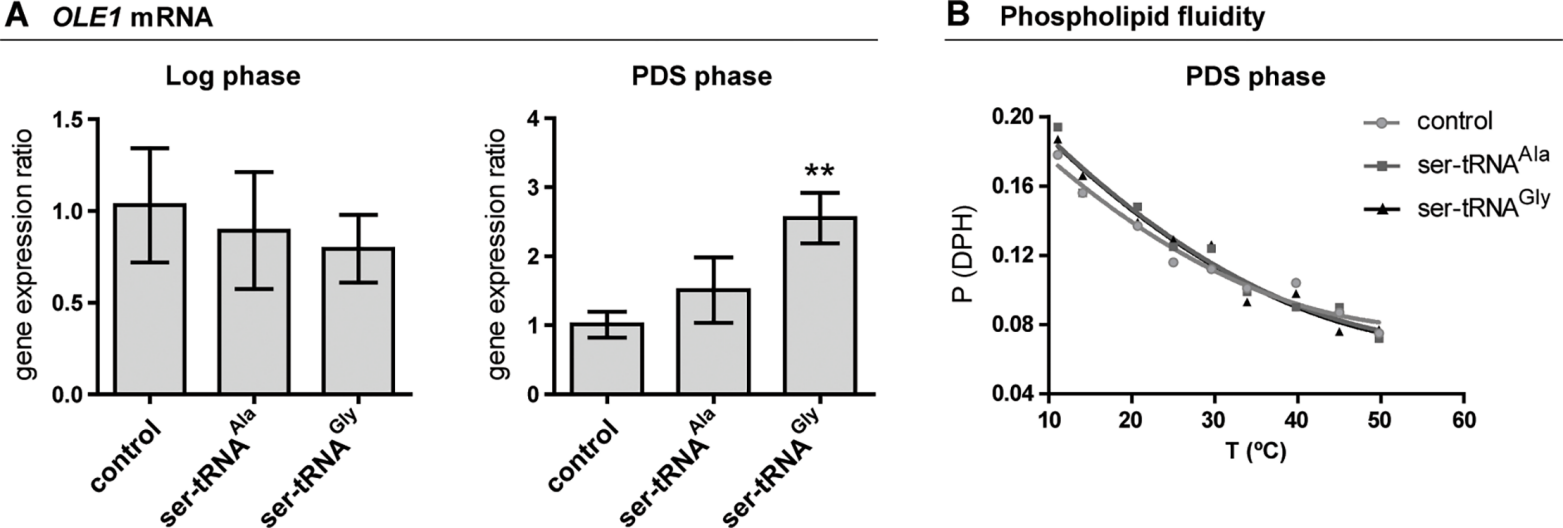
Membrane fluidity is not altered in PDS phase despite the upregulation of the Δ9 fatty acid desaturase-coding gene. **(**A) Quantitative real-time PCR analysis of expression levels of *OLE1* gene in logarithmic and PDS phases were assessed. Target/reference ratios were calculated using the mathematical model determined by Pfaffl (2001) [65]. Actin was used as the reference gene. Data are expressed as mean values ± standard deviation of three biological replicates. Statistical analysis was performed by one-way analysis of variance (ANOVA) followed by Dunnett's multiple comparison test (**P<0.01). (B) Membrane fluidity was qualitatively assessed through determination of the thermograms of fluorescence polarization of DPH in liposomes prepared with the PL extracts of *S*. *cerevisiae* strains. Liposomes were prepared with PL extracts and incubated with DPH. The depicted graph represents 1*n* and is illustrative of the observations in two biological replicates.

The reduction of MUFA relative abundance and the apparent absence of oxidative damage led us to hypothesize that the desaturation process could be altered in the mistranslating strains. In *S*. *cerevisiae*, desaturation of the bond between carbons 9 and 10 of the palmitic and stearic acids is carried out by the Δ9 fatty acid desaturase (Ole1p). This enzyme is thus essential to cell homeostasis, namely for membrane adaptation to different stimuli such as carbon source, temperature, exogenous FA supplementation, metal ions and oxygen levels. Hence, *OLE1* expression is tightly regulated [67]. We analyzed the transcripts of this gene in both growth phases, seeking to shed light on our observations. In logarithmic phase, mRNA-*OLE1* levels were similar for all strains, but in PDS phase the strain mistranslating Gly codons up-regulated *OLE1* expression by 1.5-fold whereas the Ala-to-Ser strain showed a similar trend (Fig 6A). Thus, it is possible that Ole1p is counteracting lipid homeostasis disturbance, leading to less changes in the PL species profiles. Consistently, changes in lipid composition in the PDS phase did not promote alterations in membrane fluidity, as assessed by fluorescence polarization of DPH in liposomes prepared from the phospholipid extracts of both *S*. *cerevisiae* strains (Fig 6B and S7 Fig).

## Discussion

### Different types of protein synthesis errors produce distinct phenotypic outcomes.

We show here that Ala-to-Ser and Gly-to-Ser misincorporations (Table 1 and S1 Table), have modest effects on fitness (S8 Fig), despite the observed reduction of protein activity (S2 Fig). This is in agreement with previous publications in which the expression and translational activity of the recombinant Ser-tRNA have been deeply characterized [20–22,27,29]. The chemical and physical characteristics of each amino acid–e.g. polarity–, their localization within protein structure, and eventual post-translational modifications certainly contribute to the toxicity differences observed in yeast. For instance, Ser-to-Leu mistranslation, the best studied case in *S*. *cerevisiae*, leads to higher toxicity likely due to the major chemical differences between Leu (hydrophobic) and Ser (polar) [21,29,30]. Curiously, the Ala-to-Ser mistranslation had more severe effects in *C*. *albicans* (e.g. growth rate) [31] than in *S*. *cerevisiae*. The substitution of Gly residues by Ser can compromise protein structure due to steric constraints, such as in Gly-rich loops [73–77]. Gly appears to be the only amino acid susceptible to myristoylation [78] but similarly to Ser, it is mostly localized in coil regions and can be equally positioned closer to either the core or the surface of proteins [79]. Conversely, Ala localizes mainly to α-helixes but is also found in coil regions [79]. More importantly, it is hydrophobic and its substitution by a polar amino acid like Ser could destabilize hydrophobic regions. Given the previous data from our lab regarding different amino acid substitutions, it is likely that the chemical/structural differences between the amino acids are the main drivers of the observed effects. Additionally, in human cell lines mistranslation was not associated with genome-wide codon frequencies but mainly with the nature of the substitution [80]. Therefore, the type of mistranslation is an important factor that determines the impact on proteome disruption, proteotoxic stress intensity, levels of stress response induction and ultimately phenotypic outcomes.

### The yeast lipidome is altered by protein synthesis errors

At the lipidome level, our results show that mistranslation affects mainly FA and lipid molecular species, without significantly disturbing overall PL class distribution (Fig 1) or total PL content (S9 Fig, see text). The PL/protein ratios were decreased by 30% in PDS phase, in both mistranslating strains, mostly due to decreased protein content. It is possible that protein synthesis rate decreased and/or protein degradation increased in response to accumulation of mistranslated proteins. Indeed, reduced protein synthesis is a common response to stress, namely oxidative, osmotic and heat stress, and also to proteotoxic stress induced by mistranslation [21,29,31,80–82]. However, recent studies show that misincorporated amino acids can either increase or decrease protein synthesis rate [21].

The yeast fatty acid composition varies according to genetic background and growth conditions [66,67], and to our knowledge the FA of the strains used here have not yet been studied. The main alterations in FA distribution in the mistranslating strains were more prominent in PDS phase than in log phase whereas the PL and TG molecular species analyzed showed greater changes in the log phase. In the latter, yeast cells increase the production of PL than into neutral lipids, such as TG, whose content only increases at the end of this growth phase [70]. We found interesting that TG species distribution was mainly altered in log phase, during which their content is expected to be low. It is estimated that translation can consume up to 50% of the cellular energy [83]. Therefore, since we are perturbing the proteome it is likely that these cells in fact spend more energy and need to mobilize TG. In the Ala-to-Ser strain, PL and TG species distribution was affected, in particular a shift from species carrying mono unsaturated fatty acids (MUFA) to those carrying saturated fatty acids (SFA) was observed, mostly in log phase. Since there was no exogenous FA supplementation, FA synthesis will rely on the FA *de novo* synthesis pathway [84]. If this process is affected, then it is likely that downstream synthesis processes are also altered.

**Fig 7:**
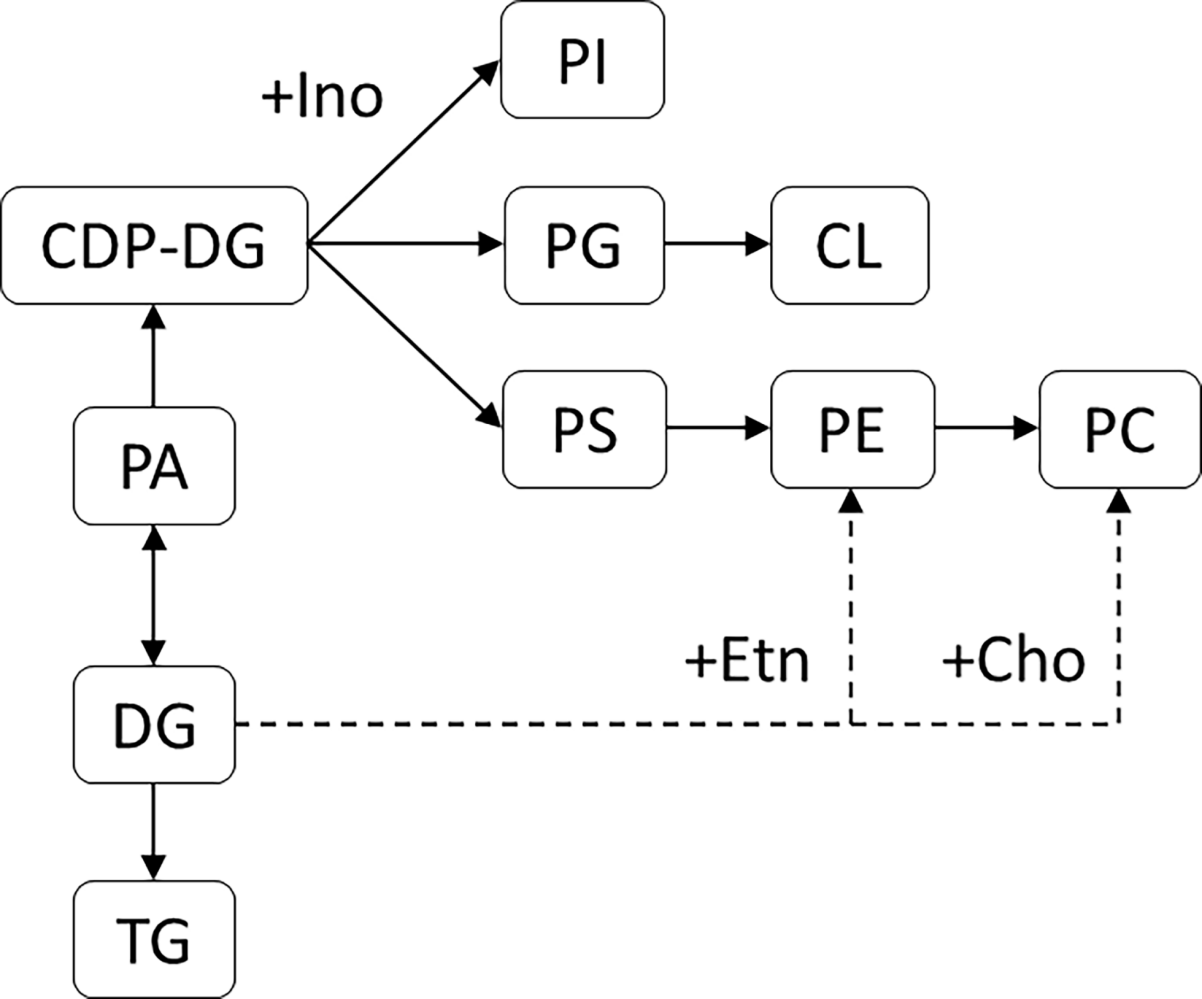
Phospholipid synthesis pathways in *S*. *cerevisiae*. This simplified scheme shows the biosynthesis routes of the PL analyzed in this study. The dashed and solid lines represent the Kennedy and the CDP-DG pathways, respectively. Cho, Choline. Etn, Ethanolamine; Ino, Inositol.

The Ala-to-Ser strain showed most of the lipid alterations detected in this study. Alterations in PL species distribution were more prominent than in the Gly-to-Ser mistranslating strain, whereas TG species distribution was less affected. In the Ala-to-Ser strain, there was a general increment in 34:1-containing PL species, mainly comprised of C16 and C18 FA, which included PA (34:1). PL synthesis is an intertwined process, in which PA is a starting point via the CDP-DG pathway. Additionally, PA is also a precursor of DG, which in turn is needed for TG synthesis, and also PE and PC synthesis via the Kennedy pathway [85] (Fig 7). Therefore, PL that derive from PA may have a comparable FA composition. In a similar way, PA-derived DG species may originate the observed alterations in TG species profiles.

In the case of the Gly-to-Ser strain, since there was no alteration in the PA profiles, the changes in TG profiles may be due to downstream enzymatic activity, either related to synthesis or turnover.

A significant variation of LPC species distribution was also only observed in the Ala-to-Ser strain. The decrease of LPC (16:1) is in line with the other lipidome-related observations (Figs 2 and 3, Table 3). A previous transcriptomics study showed that expression of *PLB1*, which codes for an enzyme involved in the remodelling of PC and PE, was upregulated in a similar misincorporation model [31]. Our observations regarding LPC and PE suggest an altered PL remodelling activity in the Ala-to-Ser strain.

Since PI-derivatives have been implicated in several signal transduction pathways [86], the changes in PI molecular species distribution, mostly noted in the Ala-to-Ser strain, may affect signal transduction. Interestingly, it is now known that the acyl composition of PA species influences the activation of the mammalian PI4P5-kinase [87], which converts PI-4-P into PI-4,5-P_2_.

### Gly/Ala mistranslation does not cause oxidative stress

Mistranslation was previously linked to increased reactive oxygen species (ROS) levels, protein oxidation and up-regulation of the antioxidant response [21,25,29,30,72,88]. Thus, we hypothesized that the observed deviations in PL and FA profiles and the decreased FA unsaturation could have resulted from oxidative damage. We measured lipid peroxidation, but the transformed strains did not significantly differ from the control strain. However, the detected hydroperoxides are unstable, and can be further oxidized or fragmented [89]. It is curious that the results were similar in both growth phases: the detected lipid hydroperoxides slightly increased in the Ala-to-Ser strain and slightly decreased in the Gly-to-Ser strain relative to the control strain. Indeed, these opposite trends between the mistranslating strains were statistically significant in log phase (S6 Fig). This indicates that each type of amino acid misincorporation is differently dealt with by the cell, which is in line with a previous phenotypic screening carried out in our laboratory [21]. Given these results, we verified if ROS levels and antioxidant enzyme activity were altered. Surprisingly, both mitochondria-specific superoxide anion and ‘total’ ROS were decreased in the Gly-to-Ser strain by 19% and 36%, respectively, relative to the control strain, and the levels of the latter were also decreased by 13% in the Ala-to-Ser strain (Fig 5B). Conversely, induction of Gly-to-Ser mistranslation in another yeast strain and in a zebrafish model of mistranslation increased ROS levels [21,72]. Regarding Ala-to-Ser misincorporation, a reduction in ROS levels was also observed in another yeast strain [21]. However, induction of Ala-to-Ser mistranslation in a zebrafish model has shown an increment in catalase activity which hinted at increased oxidative stress [72].

The decrease in ROS levels, particularly in the Gly-to-Ser strain, is consistent with the lower lipid peroxidation levels observed in this strain compared to the control strain. The observation that the activities of the antioxidant defense enzymes (Fig 5C) were unaltered points to undisturbed redox homeostasis and indicates that it is unlikely that the detected changes in ROS levels are related to upregulation of antioxidant enzymatic activity.

### Mistranslation upregulates the *OLE1* gene

We also hypothesized that the desaturation of FA, part of the FA synthesis process, could be altered by mistranslation. In yeast the Δ-9 fatty acid desaturase (Ole1p) is the only known enzyme able to generate MUFA from SFA. Since there is no evidence of post-translational modifications of Ole1p, such as the need for activation, and regulation of its expression occurs mainly at the transcriptional level with a contribution of mRNA stability [67], an increment in mRNA expression should reflect a direct demand for this enzyme. In fact, it was seen elsewhere that overexpression of this gene upregulated transcript levels by 40% [90] and resulted In accumulation of total MUFA, mainly C18:1, in detriment to SFA [90–92].

It was surprising that only the Gly-to-Ser strain upregulated *OLE1* expression because its PL profiles are barely altered in both growth phases. One possible explanation is that the acceleration of desaturation by Ole1p contributes to the re-establishment of lipid homeostasis. We have surveyed fluidity but no such alteration was observed (Fig 6B and S7 Fig). Additionally, PC species profiles were not at all or were the least altered (Figs 2 and 3, respectively) in our data set, and this class is a major constituent of cell membranes [93]. These observations were however true for both strains and not only the Gly-to-Ser. Alternatively, Ole1p may be mistranslated and degraded since its sequence has GGA and GCA codons, which may lead to misfolded Ole1p. It is possible that the Gly codon may be generally less frequent, or less present in conserved sequences/motifs, than the Ala codon on mRNA molecules that code for proteins related to synthesis/metabolism of membrane lipids. It should be noted however, that the overall changes in the lipid profiles observed here do not necessarily reflect alterations in the membranes of specific cell compartments, in which lipid profiles may vary, e.g. in opposite ways, as seen in the mitochondrial lipid profiles of our strains (unpublished) and hence we are observing the averaged profiles of all compartment/organelle profiles.

## Conclusion

Our study shows that Ser misincorporation at Gly and Ala sites on a proteome-wide scale disturbs lipid homeostasis. Fatty acid profiles were altered, including C16:0 and C16:1, in both growth phases and mistranslating strains; PL profiles were mostly altered in the Ala-to-Ser strain, whereas TG profiles were mostly changed in the other strain. The *OLE1* gene was upregulated in the Gly-to-Ser strain but membrane fluidity alterations were not observed, suggesting that the changes in lipid profiles and gene upregulation were not sufficient to change fluidity or that Ole1p is counter-acting lipid homeostasis perturbation. Moreover, oxidative stress did not contribute to the observed effects. Overall, the observations regarding the lipidome suggest that these specific amino acid exchanges are well tolerated by yeast, which is in line with the reduction in fitness observed. The mechanisms by which mistranslation alters lipids profiles remain to be elucidated. One possibility is that the alterations in lipid composition may be partly due to accumulation of misfolded proteins in the endoplasmic reticulum and consequent triggering of the unfolded protein response. This possibility should be clarified in future studies.

## Supporting information

S1 Fig The mutant tRNA_CGA_^Ser^ is expressed in the host strains. 25 μg of total RNA were fractionated on polyacrylamide gel. The mutated tRNA_CGA_^Ser^ was detected using a ɣ-^32^P-ATP-tRNA_CGA_^Ser^ probe. Ca corresponds to total RNA purified from *C*. *albicans*; EV, empty vector; Ser, native *C*. *albicans* tRNA_CGA_^Ser^ transformed into *S*. *cerevisiae*. Membranes were exposed to a K-screen and were visualized using a Bio-Rad Molecular Imager FX.
(TIF)

S2 Fig Mistranslation decreases protein stability. *E*. *coli* β-galactosidase (β-gal) was co-expressed in yeast cells with pRS315, pUA268 and pUA269. β-gal expression (A, B) was verified by western blot with an anti-β-gal antibody. Alcohol dehydrogenase (ADH) was used as loading control. The blot image of 1n represents the observations made for the set of biological replicates (A). β-gal activity (B) was measured prior to protein denaturation by heat. Data represent the mean ± standard deviation of a minimum of three biological replicates. Statistical significance was determined by one-way analysis of variance (ANOVA), followed by Bonferroni’s multiple comparison test (*P<0.05, **P<0.01).
(TIF)

S3 Fig LC-MS analysis of PL molecular species in positive (as [M+H]^+^ ions) and negative mode (as [M-H]^-^ ions) from logarithmic phase. Relative abundances of PA, PE, PS, LPC and PI molecular species in logarithmic phase are altered. Data is normalized against the sum of the reconstructed areas considered for each class and presented as mean ± standard deviation of three biological replicates. Statistical analysis was performed by two-way analysis of variance (ANOVA) followed by Bonferroni's multiple comparison test (***P<0.001 **P<0.01; *P<0.05).
(TIF)

S4 Fig LC-MS analysis of PL molecular species in positive (as [M+H]^+^ ions) and negative mode (as [M-H]^-^ ions) from post-diauxic shift phase. Relative abundances of PA, PE, PC and LPC molecular species in post-diauxic shift phase are altered. Data is normalized against the sum of the reconstructed areas considered for each class and presented as mean ± standard deviation of three biological replicates. Statistical analysis was performed by two-way analysis of variance (ANOVA) followed by Bonferroni's multiple comparison test (***P<0.001 **P<0.01; *P<0.05).
(TIF)

S5 Fig LC-MS analysis of TG molecular species in positive mode (as [M+NH4]^+^ ions) from logarithmic and post-diauxic shift phases. Relative abundances of triacylglycerol molecular species in logarithmic (A) and post-diauxic shift phase (B) were altered. Data is normalized against the sum of the reconstructed areas considered for each phase and presented as mean ± standard deviation of three biological replicates. Statistical analysis was performed by two-way analysis of variance (ANOVA) followed by Bonferroni's multiple comparison test (***P<0.001 **P<0.01; *P<0.05).
(TIF)

S6 Fig Quantification of lipid hydroperoxides. Lipid extracts from logarithmic phase were assayed with the FOX II assay. Data were normalized against the averaged values of the control strain and are presented as mean ± standard deviation of duplicates of three biological replicates. Statistical significance in all experiments was determined by one-way analysis of variance (ANOVA), followed by Bonferroni’s multiple comparison test (*P<0.05).
(TIF)

S7 Fig Thermograms of fluorescence polarization (P) of DPH. Liposomes were prepared with PL extracts from PDS (A) and logarithmic (B) phases. Membrane fluidity was qualitatively assessed in two biological replicates in each condition. Each depicted graph represents 1*n*.
(TIF)

S8 Fig Growth curves and relative growth rates. The optical density (A) of yeast cells transformed with pRS315, pUA268 and pUA269 was measured until stationary phase. Growth rates (B) were determined in the logarithmic phase and normalized to the rate of the control strain. Data represent the mean ± standard deviation of three biological replicates. Statistical significance was determined by one-way analysis of variance (ANOVA), followed by Dunnett’s multiple comparison test (*P<0.05, **P<0.01).
(TIF)

S9 Fig Phospholipid-to-protein ratios are only altered in PDS phase. Total PL were quantified after lipid extraction and normalized to the previously determined protein concentration for both logarithmic (A) and post-diauxic shift (B) phases. Data were normalized against the averaged values of the control strain and presented as mean ± standard deviation of three biological replicates. Statistical analysis was performed by two-way analysis of variance (ANOVA) followed by Bonferroni's multiple comparison test (*P<0.05).
(TIF)

S10 Fig Examples of spectra obtained for each misincorporating strain. The identified ions and corresponding amino acids are displayed in the tables below each spectrum. The spectra on the left represent the peptide with misincorporation, whereas on the right side, the same peptide is shown unaltered.
(PPTX)

S1 Table Relative frequencies of Ser misincorporation at Ala and Gly sites. (DOCX)

S2 Table Lipid species identified in the LC-MS analysis of the total lipid extracts in positive and negative modes. The complete list of TG, PC and LPC molecular species identified in positive mode, and PS, PG, PE, LPE, PI, IPC, MIPC and PA molecular species identified in the negative mode, in MS and MS/MS spectra, are annotated. Data are presented as *m/z* values (ratios of mass to charge) plus the respective sums of carbon atoms (C) and double bonds (N). In bold are the very low abundant yeast species identified only in the MS spectra, that have been described elsewhere [68–70].
(DOCX)

S3 Table Peptides with Ala→Ser (A) and Gly→Ser (B) misincorporations detected by LC-MS/MS.
(DOCX)

S1 Protocol Determination of β-galactosidase activity and quantity. (PDF)

S2 Protocol Detection of tRNA by northern blot. (PDF)

## Abbreviations

CDP-DG: cytidine diphosphate–diacylglycerol
CL: cardiolipin
DHE: dihydroethidium
DHR: dihydrorhodamine 123
ESI-MS: electrospray-mass spectrometry
FA: fatty acid
GC: gas chromatography
IPC: inositol-phosphoceramide
MIPC: mannosyl-inositol-phosphoceramide
MUFA: monounsaturated FA
LC-MS: liquid chromatography -mass spectrometry
LPC: lysophosphatidylcholine
ONPG: o-nitrophenyl-β-D-galactopyranoside
PA: phosphatidic acid
PC: phosphatidylcholine
PDS: post-diauxic shift
PE: phosphatidylethanolamine
PG: phosphatidylglycerol
PI: phosphatidylinositol
PL: phospholipid
PS: phosphatidylserine
ROS: reactive oxygen species
SP: sphingolipid
TG: triacylglycerol
TLC: thin layer chromatography
tRNA: transfer RNA
