# Supplementary figures and images for: Errors in protein synthesis increase the level of saturated fatty acids and affect the overall lipid profiles of yeast
Source: PLoS One. 2018 Aug 27;13(8):e0202402. doi: 10.1371/journal.pone.0202402 (PMC6110467; doi:10.1371/journal.pone.0202402)

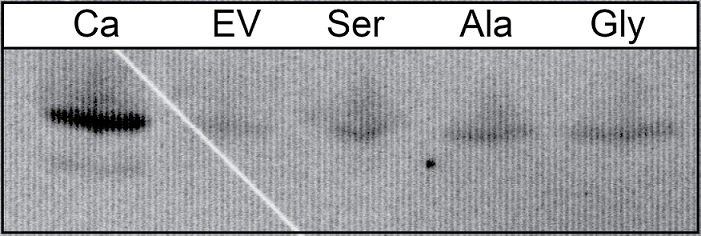

Supplement: S1 Fig — 25 μg of total RNA were fractionated on polyacrylamide gel. The mutated tRNACGASer was detected using a ɣ-32P-ATP-tRNACGASer probe. Ca corresponds to total RNA purified from C. albicans; EV, empty vector; Ser, native C. albicans tRNACGASer transformed into S. cerevisiae. Membranes were exposed to a K-screen and were visualized using a Bio-Rad Molecular Imager FX. (TIF) [file pone.0202402.s001.tif]

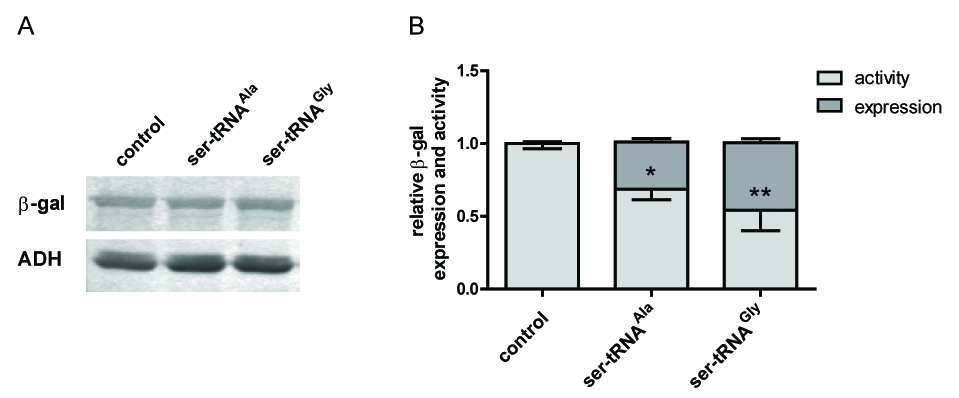

Supplement: S2 Fig — E. coli β-galactosidase (β-gal) was co-expressed in yeast cells with pRS315, pUA268 and pUA269. β-gal expression (A, B) was verified by western blot with an anti-β-gal antibody. Alcohol dehydrogenase (ADH) was used as loading control. The blot image of 1n represents the observations made for the set of biological replicates (A). β-gal activity (B) was measured prior to protein denaturation by heat. Data represent the mean ± standard deviation of a minimum of three biological replicates. Statistical significance was determined by one-way analysis of variance (ANOVA), followed by Bonferroni’s multiple comparison test (*P<0.05, **P<0.01). (TIF) [file pone.0202402.s002.tif]

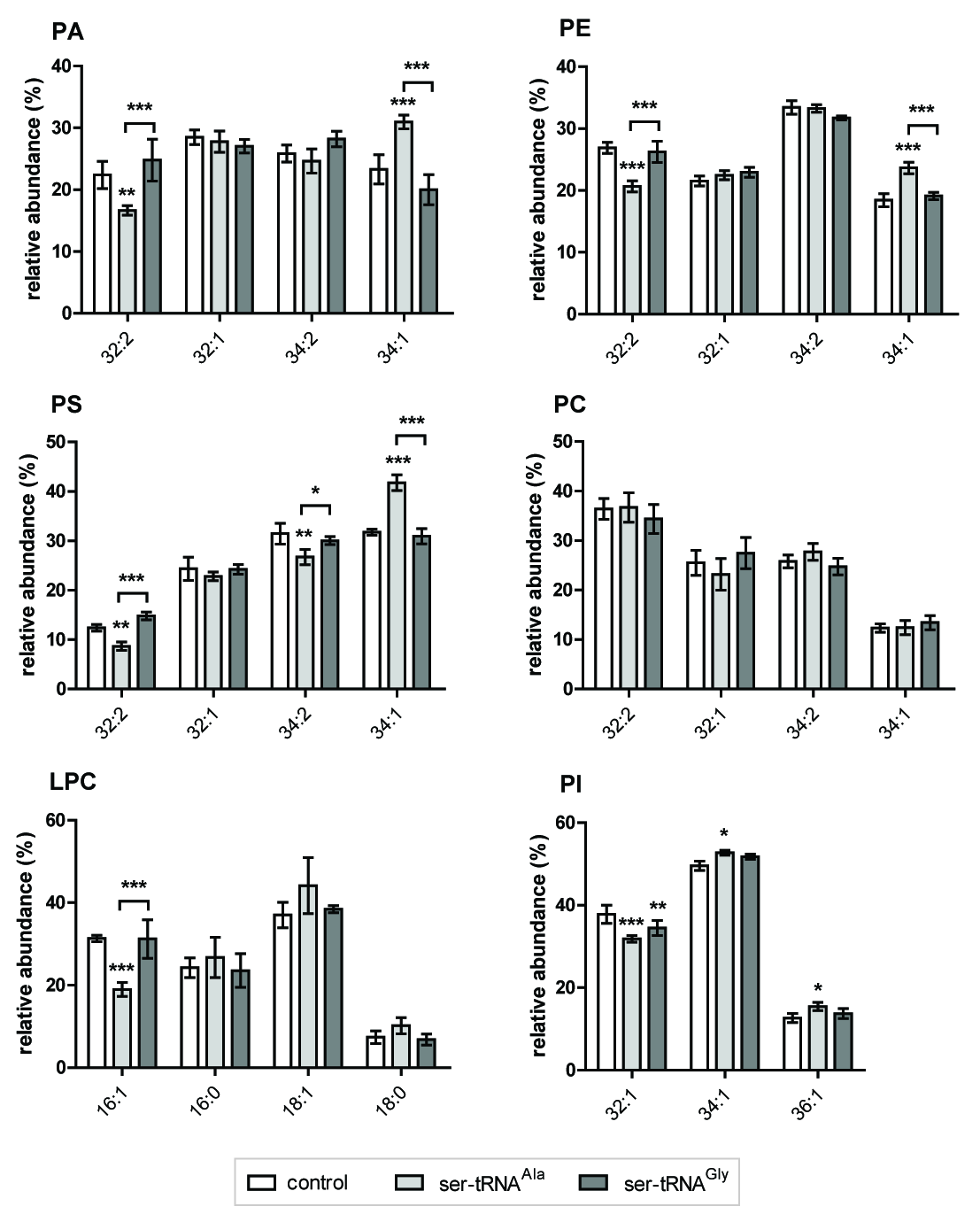

Supplement: S3 Fig — Relative abundances of PA, PE, PS, LPC and PI molecular species in logarithmic phase are altered. Data is normalized against the sum of the reconstructed areas considered for each class and presented as mean ± standard deviation of three biological replicates. Statistical analysis was performed by two-way analysis of variance (ANOVA) followed by Bonferroni's multiple comparison test (***P<0.001 **P<0.01; *P<0.05). (TIF) [file pone.0202402.s003.tif]

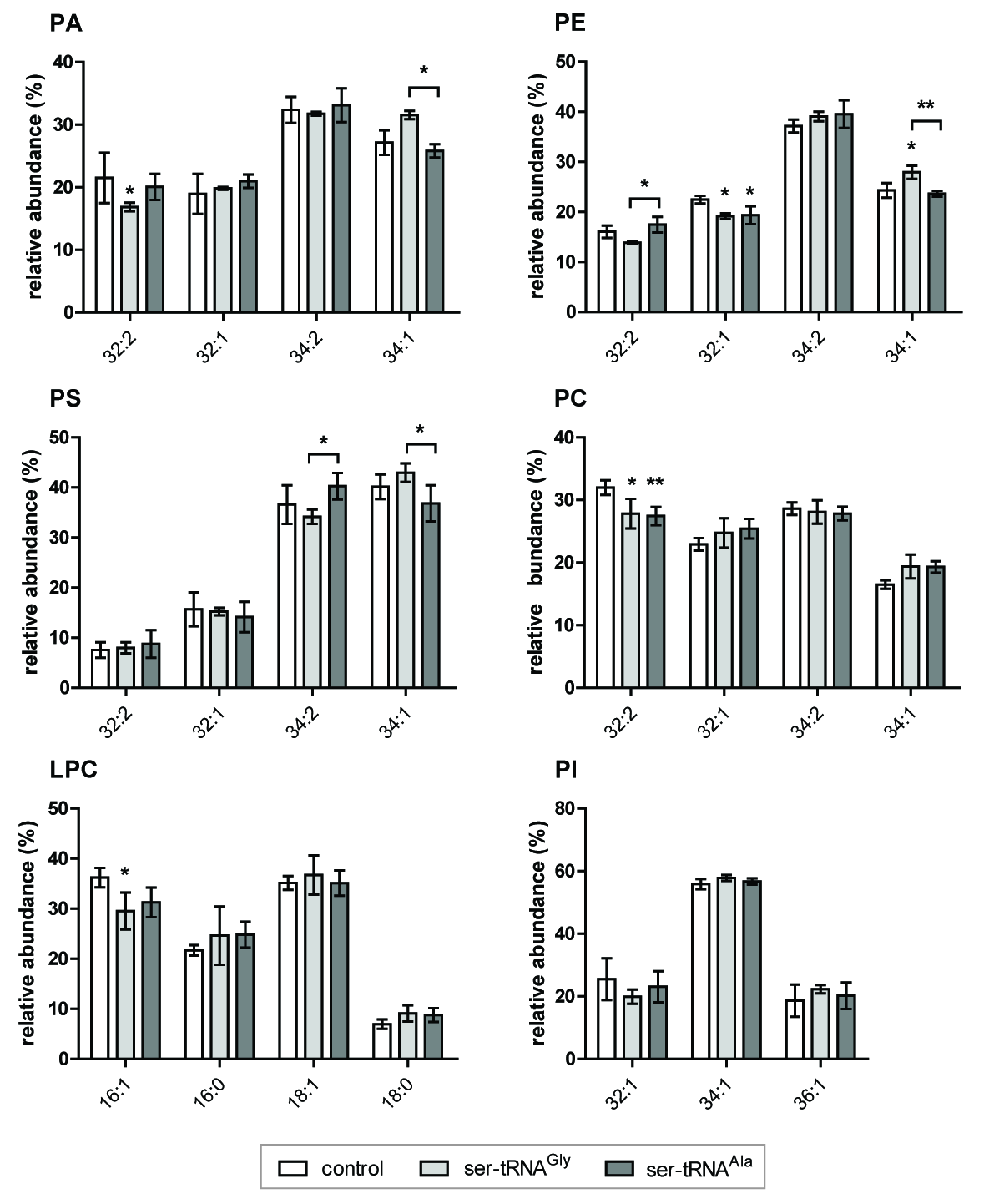

Supplement: S4 Fig — Relative abundances of PA, PE, PC and LPC molecular species in post-diauxic shift phase are altered. Data is normalized against the sum of the reconstructed areas considered for each class and presented as mean ± standard deviation of three biological replicates. Statistical analysis was performed by two-way analysis of variance (ANOVA) followed by Bonferroni's multiple comparison test (***P<0.001 **P<0.01; *P<0.05). (TIF) [file pone.0202402.s004.tif]

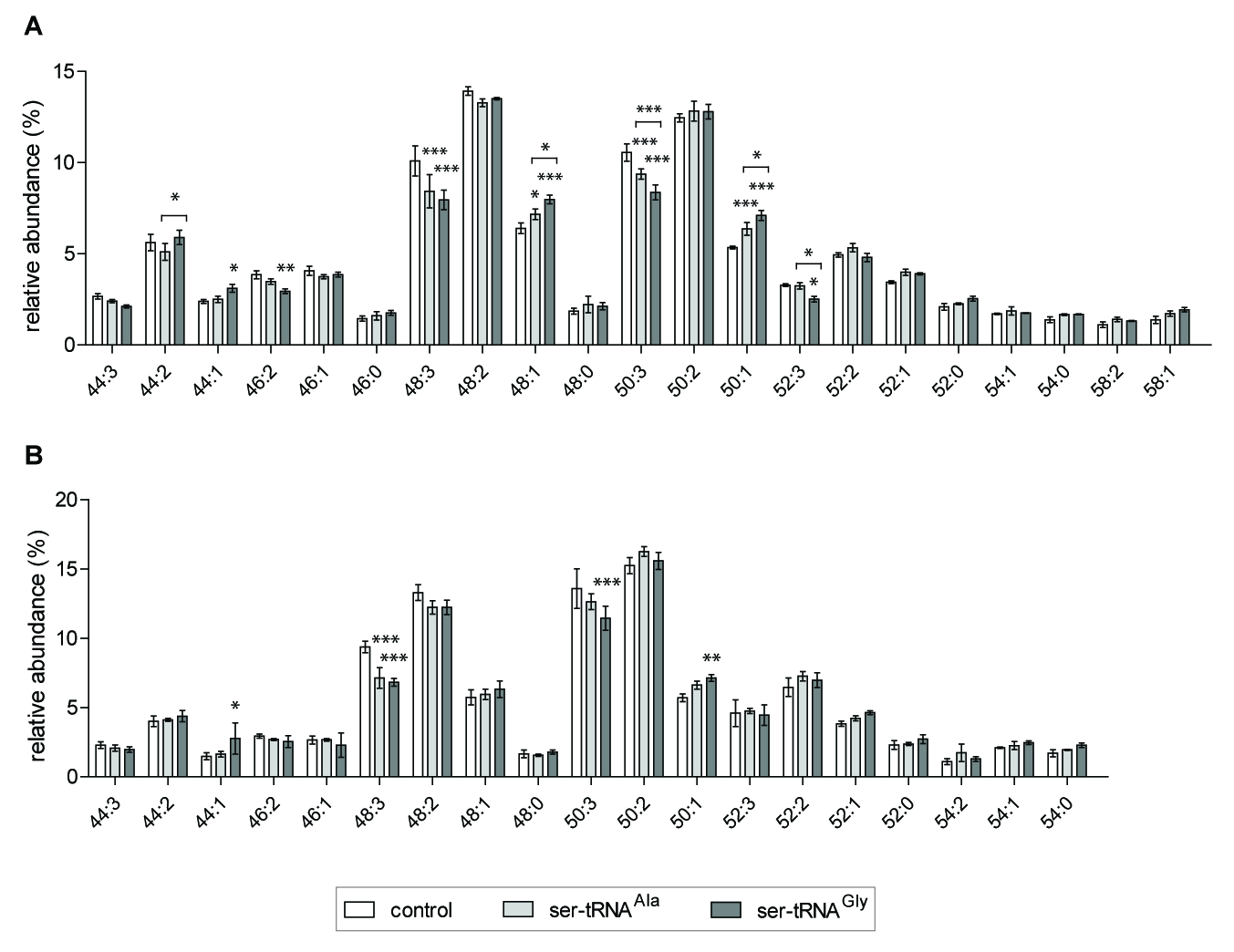

Supplement: S5 Fig — Relative abundances of triacylglycerol molecular species in logarithmic (A) and post-diauxic shift phase (B) were altered. Data is normalized against the sum of the reconstructed areas considered for each phase and presented as mean ± standard deviation of three biological replicates. Statistical analysis was performed by two-way analysis of variance (ANOVA) followed by Bonferroni's multiple comparison test (***P<0.001 **P<0.01; *P<0.05). (TIF) [file pone.0202402.s005.tif]

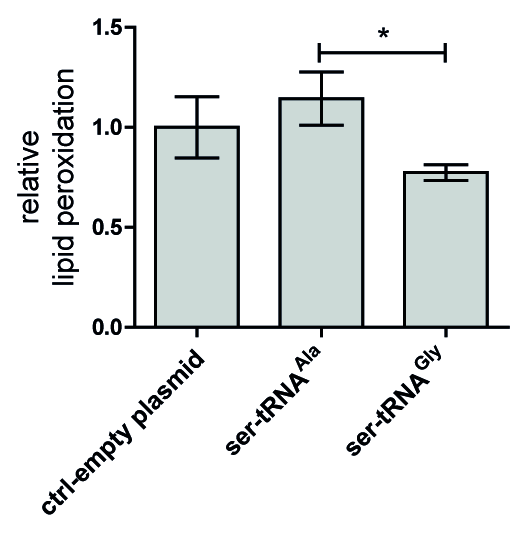

Supplement: S6 Fig — Lipid extracts from logarithmic phase were assayed with the FOX II assay. Data were normalized against the averaged values of the control strain and are presented as mean ± standard deviation of duplicates of three biological replicates. Statistical significance in all experiments was determined by one-way analysis of variance (ANOVA), followed by Bonferroni’s multiple comparison test (*P<0.05). (TIF) [file pone.0202402.s006.tif]

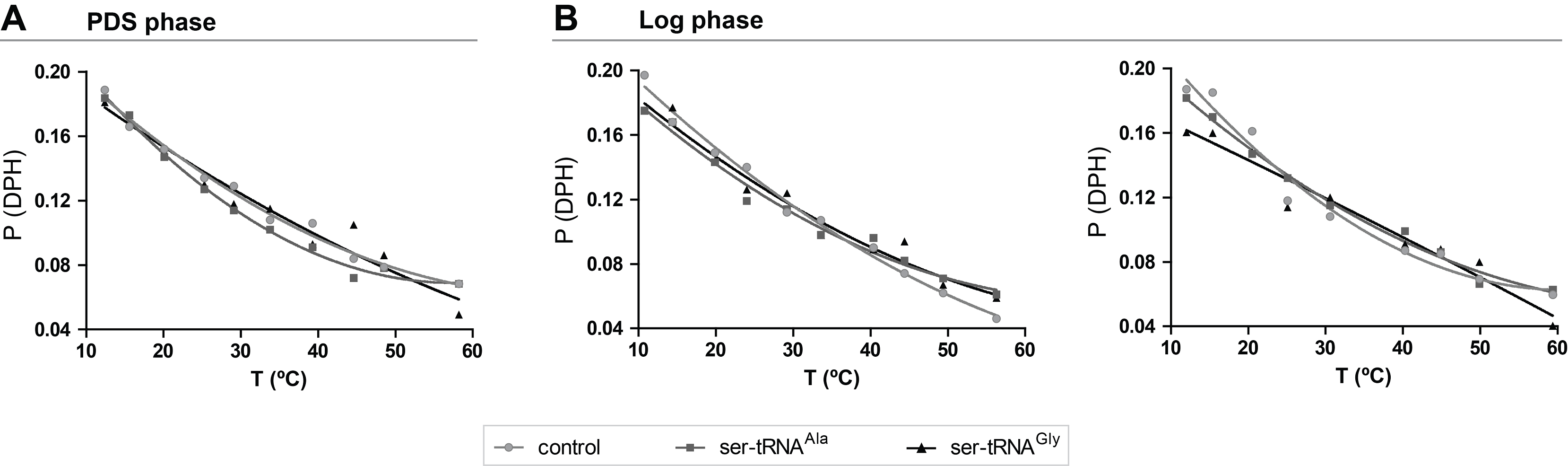

Supplement: S7 Fig — Liposomes were prepared with PL extracts from PDS (A) and logarithmic (B) phases. Membrane fluidity was qualitatively assessed in two biological replicates in each condition. Each depicted graph represents 1n. (TIF) [file pone.0202402.s007.tif]

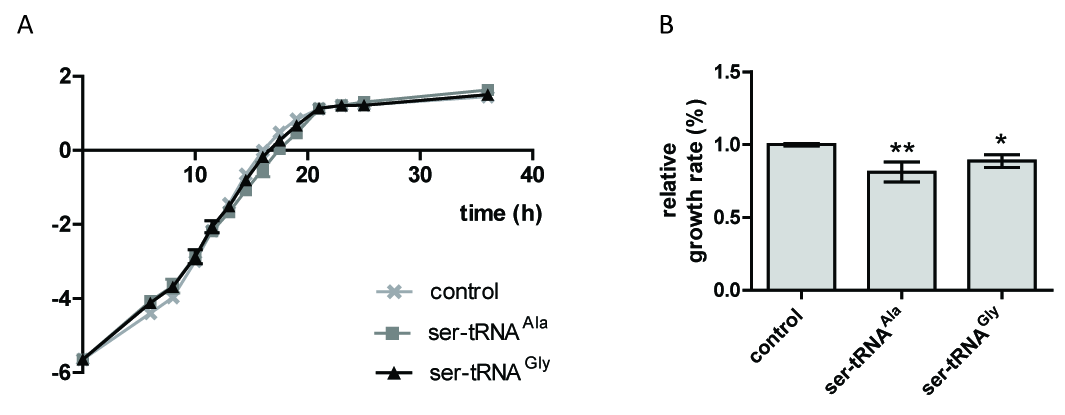

Supplement: S8 Fig — The optical density (A) of yeast cells transformed with pRS315, pUA268 and pUA269 was measured until stationary phase. Growth rates (B) were determined in the logarithmic phase and normalized to the rate of the control strain. Data represent the mean ± standard deviation of three biological replicates. Statistical significance was determined by one-way analysis of variance (ANOVA), followed by Dunnett’s multiple comparison test (*P<0.05, **P<0.01). (TIF) [file pone.0202402.s008.tif]

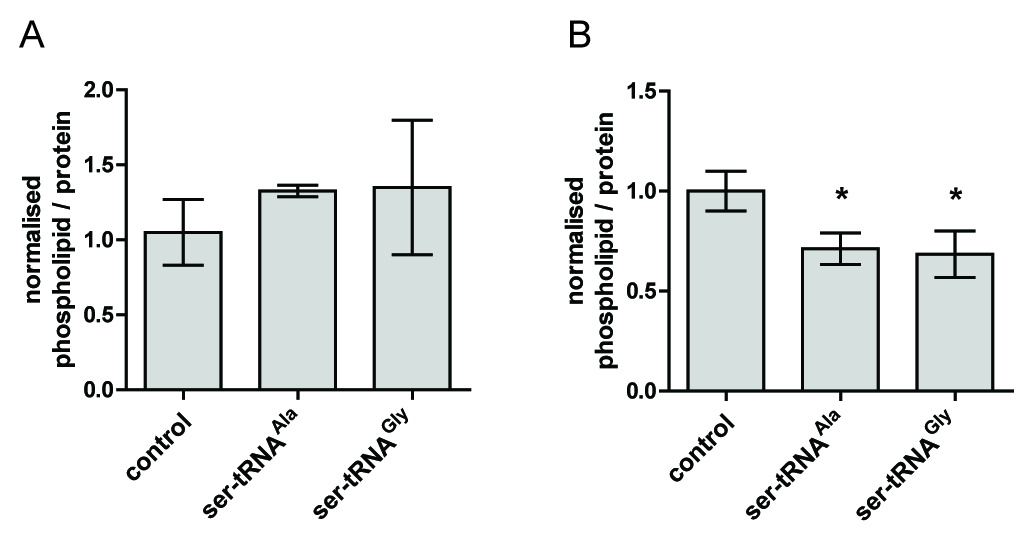

Supplement: S9 Fig — Total PL were quantified after lipid extraction and normalized to the previously determined protein concentration for both logarithmic (A) and post-diauxic shift (B) phases. Data were normalized against the averaged values of the control strain and presented as mean ± standard deviation of three biological replicates. Statistical analysis was performed by two-way analysis of variance (ANOVA) followed by Bonferroni's multiple comparison test (*P<0.05). (TIF) [file pone.0202402.s009.tif]
