# Supplementary material for: Errors in protein synthesis increase the level of saturated fatty acids and affect the overall lipid profiles of yeast
Source: PLoS One. 2018 Aug 27;13(8):e0202402. doi: 10.1371/journal.pone.0202402 (PMC6110467; doi:10.1371/journal.pone.0202402)
Supplement: S10 Fig — The identified ions and corresponding amino acids are displayed in the tables below each spectrum. The spectra on the left represent the peptide with misincorporation, whereas on the right side, the same peptide is shown unaltered. (PPTX) [file pone.0202402.s010.pptx]

## Slide 1
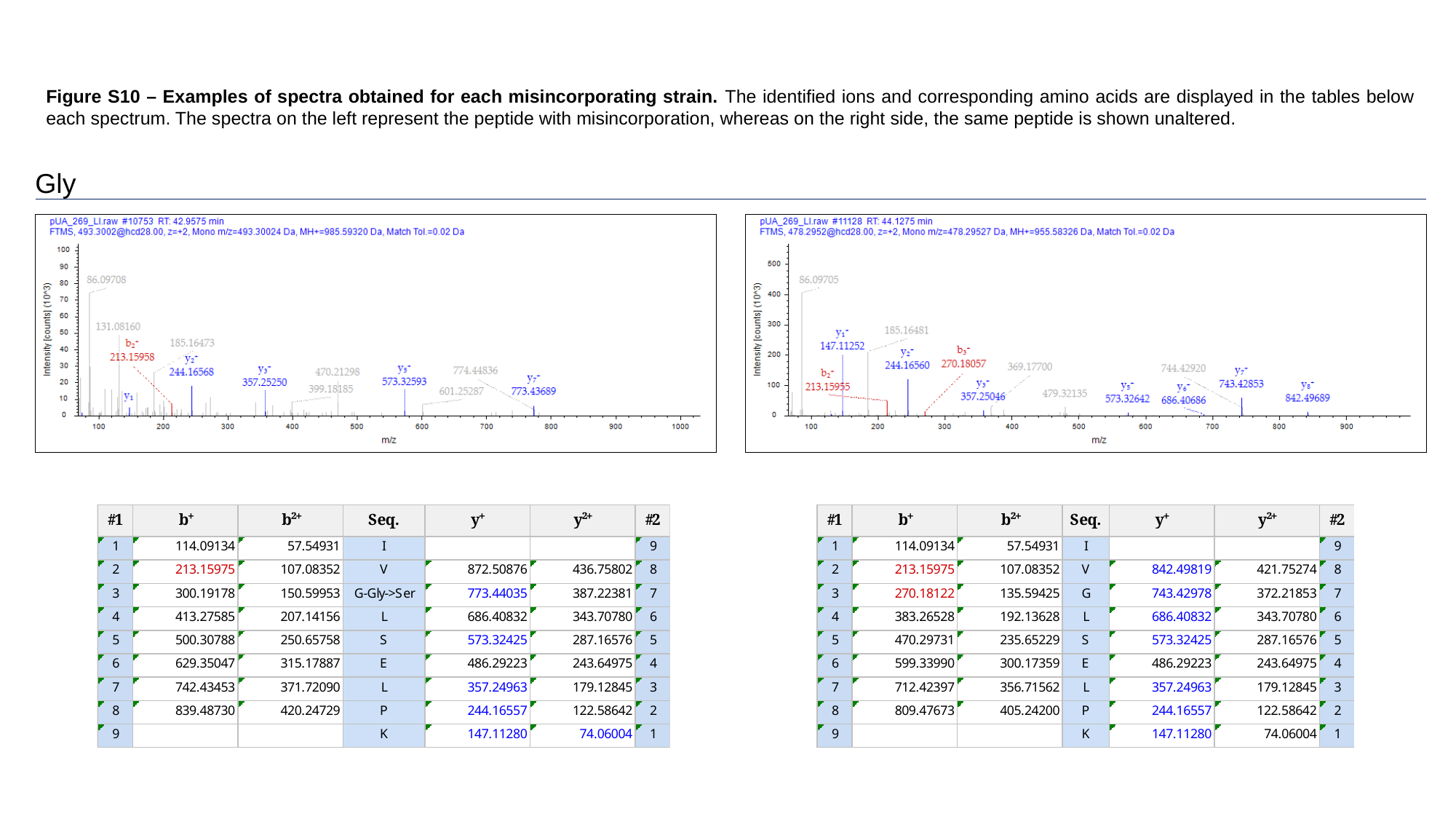

Figure S10 – Examples of spectra obtained for each misincorporating strain. The identified ions and corresponding amino acids are displayed in the tables below each spectrum. The spectra on the left represent the peptide with misincorporation, whereas on the right side, the same peptide is shown unaltered.
Gly

## Slide 2
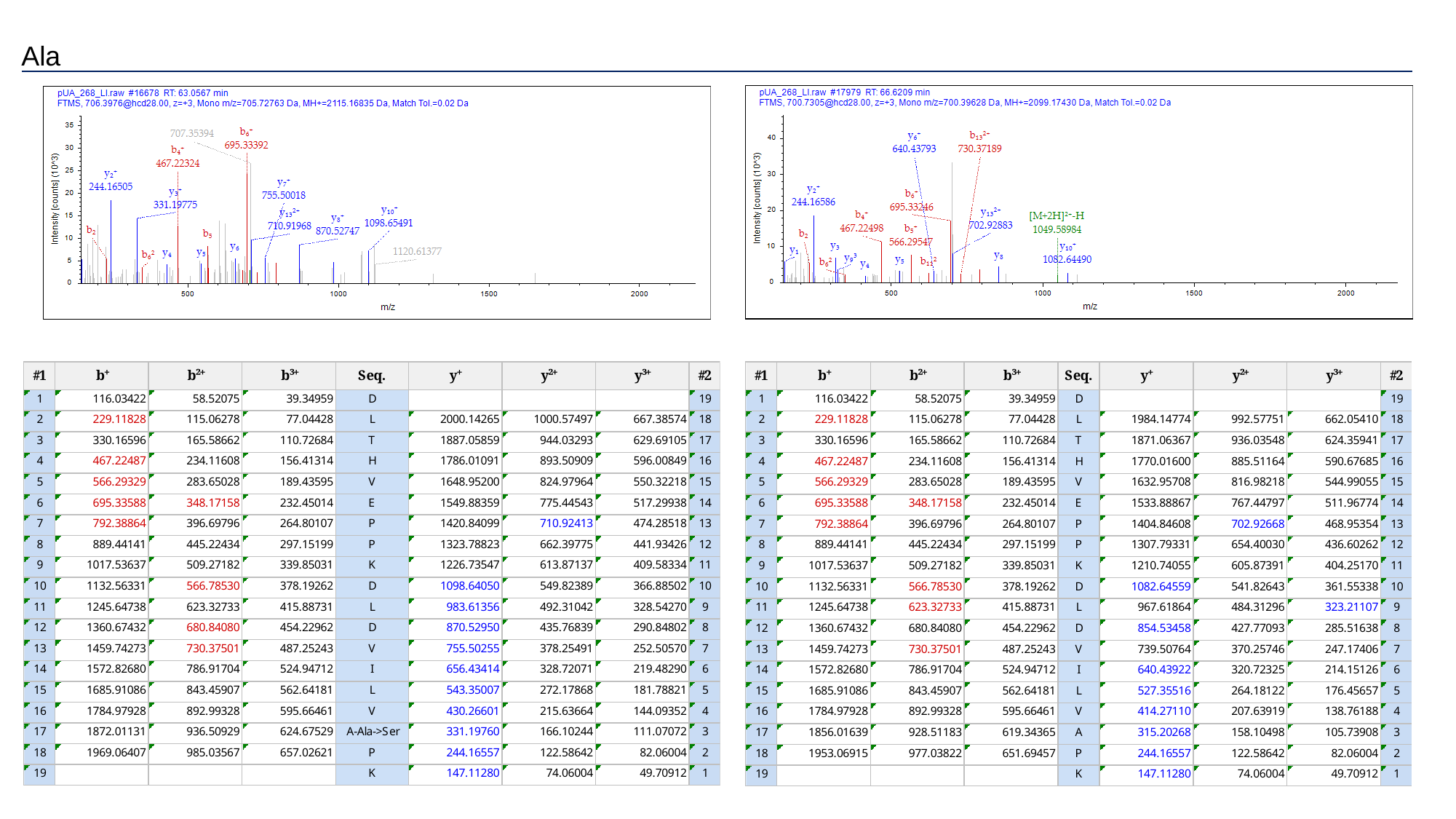

Ala
