# Supplementary material for: Errors in protein synthesis increase the level of saturated fatty acids and affect the overall lipid profiles of yeast
Source: PLoS One. 2018 Aug 27;13(8):e0202402. doi: 10.1371/journal.pone.0202402 (PMC6110467; doi:10.1371/journal.pone.0202402)
Supplement: S1 Table — (DOCX) [file pone.0202402.s011.docx]

**S1 Table.** Relative frequencies of serine misincorporation at Ala and Gly sites. Identification was achieved through MS analysis of a peptide fraction. * codons decoded by the mutant tRNAs; NA, not applicable.

| Ser misincorporation frequency | | | |  |  |
| --- | --- | --- | --- | --- | --- |
|  |  | Control | Ser-tRNA^Ala^ | | Ser-tRNA^Gly^ |
| Ala | GCA* | 0,006 | 0,015 | | NA |
|  | GCG* | 0,059 | 0,114 | | NA |
|  | GCT | 0,021 | 0,067 | | NA |
|  | GCC | 0,000 | 0,000 | | NA |
| Gly | GGA* | 0,000 | NA | | 0,002 |
|  | GGG | 0,059 | NA | | 0,000 |
|  | GGT | 0,016 | NA | | 0,045 |
|  | GGC | 0,002 | NA | | 0,006 |
