# Supplementary material for: Errors in protein synthesis increase the level of saturated fatty acids and affect the overall lipid profiles of yeast
Source: PLoS One. 2018 Aug 27;13(8):e0202402. doi: 10.1371/journal.pone.0202402 (PMC6110467; doi:10.1371/journal.pone.0202402)
Supplement: S2 Table — The complete list of TG, PC and LPC molecular species identified in positive mode, and PS, PG, PE, LPE, PI, IPC, MIPC and PA molecular species identified in the negative mode, in MS and MS/MS spectra, are annotated. Data are presented as m/z values (ratios of mass to charge) plus the respective sums of carbon atoms (C) and double bonds (N). In bold are the very low abundant yeast species identified only in the MS spectra, that have been described elsewhere [68–70]. (DOCX) [file pone.0202402.s012.docx]

S2 Table. Lipid species identified in the HILIC-MS analysis of the total lipid extracts in positive and negative modes. The complete list of TG, PC and LPC molecular species identified in positive mode, and PS, PG, PE, LPE, PI, IPC, MIPC and PA molecular species identified in the negative mode, in MS and MS/MS spectra, are annotated. Data are presented as *m/z* values (ratios of mass to charge) plus the respective sums of carbon atoms (C) and double bonds (N). In bold are the very low abundant yeast species identified only in the MS spectra, that have been described elsewhere [67–69].

| TG | m/z  [M+NH_4_]^+^ | C:N |  | PC | m/z  [M+H]^+^ | C:N |  | LPC | | m/z  [M+H]^+^ | | | C:N | | |
| --- | --- | --- | --- | --- | --- | --- | --- | --- | --- | --- | --- | --- | --- | --- | --- |
|  | 736 | 42:2 |  |  | 648 | 26:1 |  |  |  | 468 | | | 14:0 | | |
|  | 738 | 42:1 |  |  | 650 | 26:0 |  |  |  | 494 | | | 16:1 | | |
|  | 740 | 42:0 |  |  | 676 | 28:1 |  |  |  | 496 | | | 16:0 | | |
|  | 764 | 44:3 |  |  | 678 | 28:0 |  |  |  | 522 | | | 18:1 | | |
|  | 766 | 44:2 |  |  | **702** | **30:2** |  |  |  | 524 | | | 18:0 | | |
|  | 768 | 44:1 |  |  | **704** | **30:1** |  |  | |  | |  | | |  |
|  | **790** | **46:3** |  |  | **706** | **30:0** |  |  | |  | |  | | |  |
|  | 792 | 46:2 |  |  | 730 | 32:2 |  |  | |  | |  | | |  |
|  | 794 | 46:1 |  |  | 732 | 32:1 |  |  | |  | |  | | |  |
|  | 796 | 46:0 |  |  | 734 | 32:0 |  |  | |  | |  | | |  |
|  | 818 | 48:3 |  |  | 758 | 34:2 |  |  | |  | |  | | |  |
|  | 820 | 48:2 |  |  | 760 | 34:1 |  |  | |  | |  | | |  |
|  | 822 | 48:1 |  |  | 786 | 36:2 |  |  | |  | |  | | |  |
|  | 846 | 50:3 |  |  | 788 | 36:1 |  |  | |  | |  | | |  |
|  | 848 | 50:2 |  |  | **790** | **36:0** |  |  | |  | |  | | |  |
|  | 850 | 50:1 |  |  |  |  |  |  | |  | |  | | |  |
|  | 874 | 52:3 |  |  |  |  |  |  | |  | |  | | |  |
|  | 876 | 52:2 |  |  |  |  |  |  | |  | |  | | |  |
|  | 878 | 52:1 |  |  |  |  |  |  | |  | |  | | |  |
|  | 880 | 52:0 |  |  |  |  |  |  | |  | |  | | |  |
|  | 902 | 54:3 |  |  |  |  |  |  | |  | |  | | |  |
|  | 904 | 54:2 |  |  |  |  |  |  | |  | |  | | |  |
|  | 906 | 54:1 |  |  |  |  |  |  | |  | |  | | |  |
|  | 908 | 54:0 |  |  |  |  |  |  | |  | |  | | |  |
|  | **932** | **56:2** |  |  |  |  |  |  | |  | |  | | |  |
|  | 934 | 56:1 |  |  |  |  |  |  |  | |  | | |  |  |
|  | **936** | **56:0** |  |  |  |  |  |  |  | |  | | |  |  |
|  | 960 | 58:2 |  |  |  |  |  |  |  | |  | | |  |  |
|  | 962 | 58:1 |  |  |  |  |  |  |  | |  | | |  |  |
|  | 964 | 58:0 |  |  |  |  |  |  |  | |  | | |  |  |
|  | 988 | 60:2 |  |  |  |  |  |  |  | |  | | |  |  |
|  | 990 | 60:1 |  |  |  |  |  |  |  | |  | | |  |  |

| PS | m/z  [M-H]^-^ | C:N |  | PG | m/z  [M-H]^-^ | C:N |  | PE | m/z  [M-H]^-^ | C:N |  | LPE | m/z  [M-H]^-^ | C:N |
| --- | --- | --- | --- | --- | --- | --- | --- | --- | --- | --- | --- | --- | --- | --- |
|  | 730 | 32:2 |  |  | **717** | **32:2** |  |  | **632** | **28:1** |  |  | 450 | 16:1 |
|  | 732 | 32:1 |  |  | 719 | 32:1 |  |  | **634** | **28:0** |  |  | 452 | 16:0 |
|  | 734 | 32:0 |  |  | 745 | 34:2 |  |  | **660** | **30:1** |  |  | 478 | 18:1 |
|  | 758 | 34:2 |  |  | 747 | 34:1 |  |  | **686** | **32:2** |  |  |  |  |
|  | 760 | 34:1 |  |  |  |  |  |  | 688 | 32:1 |  |  |  |  |
|  |  |  |  |  |  |  |  |  | 714 | 34:2 |  |  |  |  |
|  |  |  |  |  |  |  |  |  | 716 | 34:1 |  |  |  |  |
|  |  |  |  |  |  |  |  |  | 742 | 36:2 |  |  |  |  |
|  |  |  |  |  |  |  |  |  | 744 | 36:1 |  |  |  |  |

| PI | m/z  [M-H]^-^ | C:N |  |  |  | m/z  [M-H]^-^ | C:N;OH |  |  | PA | m/z  [M-H]^-^ | C:N |
| --- | --- | --- | --- | --- | --- | --- | --- | --- | --- | --- | --- | --- |
|  | 725 | 26:0 |  |  | IPC | 952 | 44:0;4 |  |  |  | 643 | 32:2 |
|  | 753 | 28:0 |  |  |  | 980 | 36:0;4 |  |  |  | 645 | 32:1 |
|  | **779** | **30:1** |  |  |  | 964 | 36:0;3 |  |  |  | 671 | 34:2 |
|  | 781 | 30:0 |  |  | MIPC | 1098 | 44:0;3 |  |  |  | 673 | 34:1 |
|  | 805 | 32:2 |  |  |  | 1114 | 44:0;4 |  |  |  | 675 | 34:0 |
|  | 807 | 32:1 |  |  |  | 1142 | 36:0;4 |  |  |  | 699 | 36:2 |
|  | 809 | 32:0 |  |  |  |  |  |  |  |  |  |  |
|  | 833 | 34:2 |  |  |  |  |  |  |  |  |  |  |
|  | 835 | 34:1 |  |  |  |  |  |  |  |  |  |  |
|  | 837 | 34:0 |  |  |  |  |  |  |  |  |  |  |
|  | 861 | 36:2 |  |  |  |  |  |  |  |  |  |  |
|  | 863 | 36:1 |  |  |  |  |  |  |  |  |  |  |
|  | 865 | 36:0 |  |  |  |  |  |  |  |  |  |  |
