# Supplementary material for: Errors in protein synthesis increase the level of saturated fatty acids and affect the overall lipid profiles of yeast
Source: PLoS One. 2018 Aug 27;13(8):e0202402. doi: 10.1371/journal.pone.0202402 (PMC6110467; doi:10.1371/journal.pone.0202402)
Supplement: S3 Table — Peptides with Ala→Ser (A) and Gly→Ser (B) misincorporations detected by LC-MS/MS. (DOCX) [file pone.0202402.s013.docx]

**S3 Table – A: Peptides with Ala→Ser misincorporation detected by LC-MS/MS.**

| **Strain** | **Uniprot ID** | **Peptide** | **Start** | **End** |
| --- | --- | --- | --- | --- |
| **Control** | P00330 | YSGVCHTDLHS(A)WHGDWPLPVK | 40 | 60 |
| **Control** | P00330 | S(A)TDGGS(A)HGVINVSVSEAAIEASTR | 235 | 258 |
| **Control** | P00330 | S(A)TDGGS(A)HGVINVSVSES(A)S(A)IES(A)STR | 235 | 258 |
| **Control** | P00330 | ANGTTVLVGMPS(A)GAK | 262 | 276 |
| **Control** | P00330 | EALDFFS(A)R | 304 | 311 |
| **Control** | P00331 | EALDFFS(A)R | 304 | 311 |
| **Control** | P00358 | KVVITAPSSTS(A)PMFVMGVNEEK | 116 | 137 |
| **Control** | P00358 | VVITS(A)PSSTS(A)PMFVMGVNEEK | 117 | 137 |
| **Control** | P00358 | IVSNS(A)SCTTNCLAPLAK | 144 | 160 |
| **Control** | P00359 | KVVITAPSSTS(A)PMFVMGVNEEK | 116 | 137 |
| **Control** | P00359 | VVITS(A)PSSTS(A)PMFVMGVNEEK | 117 | 137 |
| **Control** | P00359 | IVSNS(A)SCTTNCLAPLAK | 144 | 160 |
| **Control** | P00359 | VINDS(A)FGIEEGLMTTVHSLTS(A)TQK | 161 | 184 |
| **Control** | P00360 | IVSNS(A)SCTTNCLAPLAK | 144 | 160 |
| **Control** | P00549 | VTDGVMVS(A)R | 256 | 264 |
| **Control** | P00560 | S(A)HSSMVGFDLPQR | 170 | 182 |
| **Control** | P00812 | HGLQTSIEDLGWSTELEPSMDES(A)QFVGK | 41 | 68 |
| **Control** | P00925 | WLTGVELS(A)DMYHSLMK | 273 | 288 |
| **Control** | P02994 | FVPSKPMCVES(A)FSEYPPLGR | 402 | 421 |
| **Control** | P04806 | LS(A)VCGIAAICQK | 395 | 406 |
| **Control** | P05150 | LYS(A)S(A)MSS(A)IDIFVNNK | 317 | 331 |
| **Control** | P05373 | MHTS(A)EFLETEPTEISSVLAGGYNHPLLR | 1 | 28 |
| **Control** | P06168 | S(A)S(A)IEDGWVPGK | 116 | 126 |
| **Control** | P06168 | RGSYVMNLLSDS(A)S(A)QSETWPS(A)IKPLLTK | 137 | 163 |
| **Control** | P06168 | RGSYVMNLLSDS(A)S(A)QSETWPS(A)IKPLLTK | 137 | 163 |
| **Control** | P06168 | GSYVMNLLSDS(A)S(A)QSETWPS(A)IKPLLTK | 138 | 163 |
| **Control** | P06168 | GCLMGGIHGMFLS(A)QYDVLR | 261 | 279 |
| **Control** | P06168 | ENGHSPSES(A)FNETVEES(A)TQSLYPLIGK | 280 | 306 |
| **Control** | P06168 | YGMDYMYDS(A)CSTTS(A)R | 307 | 321 |
| **Control** | P06169 | MSS(A)NISETTS(A)MITDIS(A)TS(A)PS(A)EIDR | 128 | 151 |
| **Control** | P06169 | LLQTPIDMSLKPNDS(A)ESEKEVIDTILALVK | 180 | 209 |
| **Control** | P07246 | YSGVCHTDLHS(A)WHGDWPLPVK | 67 | 87 |
| **Control** | P07264 | SDDTPS(A)KPSSSGMKPFLTLEGISAPLDK | 533 | 560 |
| **Control** | P07703 | NHFIFNVESAGS(A)MTPEEIFFK | 296 | 316 |
| **Control** | P07834 | LVS(A)NMDRSELSDLGTLIK | 250 | 267 |
| **Control** | P08524 | LVEELNS(A)SLLS(A)YGMPK | 19 | 34 |
| **Control** | P08524 | VPEVGEIAINDS(A)FMLEAAIYK | 118 | 138 |
| **Control** | P08566 | MQQRPIS(A)PLVDSLR | 530 | 543 |
| **Control** | P0CX24 | VS(A)S(A)VETLYQDMS(A)S(A)R | 100 | 113 |
| **Control** | P10591 | NQAS(A)MNPSNTVFDAK | 55 | 69 |
| **Control** | P10592 | NQS(A)S(A)MNPS(A)NTVFDS(A)K | 55 | 69 |
| **Control** | P10592 | SEVFSTYS(A)DNQPGVLIQVFEGERA | 422 | 444 |
| **Control** | P10664 | FVIWTES(A)S(A)FTK | 247 | 257 |
| **Control** | P13663 | EFMES(A)GIAIVSNAK | 96 | 109 |
| **Control** | P15646 | LAAGIMGGLDELFIS(A)PGK | 151 | 168 |
| **Control** | P15646 | LAS(A)GIMGGLDELFIAPGKK | 151 | 168 |
| **Control** | P15646 | MLIGMVDCVFS(A)DVAQPDQAR | 232 | 251 |
| **Control** | P17255 | MKEILSNS(A)EELEQVVQLVGK | 940 | 959 |
| **Control** | P20051 | DGGVSIS(A)YIMPNLQPPITTLDR | 32 | 53 |
| **Control** | P20459 | SS(A)EDMSTEQMQVK | 207 | 219 |
| **Control** | P22202 | SEVFSTYS(A)DNQPGVLIQVFEGERA | 422 | 444 |
| **Control** | P28272 | DAFEHLLCGS(A)SMLQIGTELQK | 259 | 279 |
| **Control** | P28834 | GQNVANPTS(A)MILSSTLMLNHLGLNEYATR | 293 | 321 |
| **Control** | P38113 | DIVGAIIKS(A)TNGGSHGVINVSVSEAAIEASTR | 230 | 261 |
| **Control** | P38113 | EALDFFS(A)R | 307 | 314 |
| **Control** | P38115 | SSSVS(A)STENIVENMLHPK | 2 | 9 |
| **Control** | P38891 | GLGVGTPSES(A)LLYVITSPVGPYYK | 170 | 193 |
| **Control** | P38891 | LGANYS(A)PCILPQLQAAK | 220 | 236 |
| **Control** | P38891 | QVS(A)QWIS(A)DIQYGR | 367 | 379 |
| **Control** | P39987 | NAVVTVPAYFNDS(A)QR | 162 | 176 |
| **Control** | P41808 | SVDIWS(A)VGCILS(A)EFYS(A)R | 230 | 246 |
| **Control** | P49626 | FVIWTES(A)S(A)FTK | 247 | 257 |
| **Control** | P53111 | FTLSTINPGFVFGPQMFS(A)DSLK | 193 | 214 |
| **Control** | Q04894 | MGADHYIS(A)TLEEGDWGEK | 222 | 239 |
| **Ser-tRNA^Ala^** | P00330 | DIVGS(A)VLK | 227 | 234 |
| **Ser-tRNA^Ala^** | P00330 | S(A)NELLINVK | 31 | 39 |
| **Ser-tRNA^Ala^** | P00330 | YSGVCHTDLHS(A)WHGDWPLPVK | 40 | 60 |
| **Ser-tRNA^Ala^** | P00330 | LPLVGGHEGS(A)GVVVGMGENVK | 61 | 81 |
| **Ser-tRNA^Ala^** | P00330 | S(A)TDGGS(A)HGVINVSVSES(A)S(A)IES(A)STR | 235 | 258 |
| **Ser-tRNA^Ala^** | P00330 | ES(A)LDFFS(A)R | 304 | 311 |
| **Ser-tRNA^Ala^** | P00331 | YSGVCHTDLHS(A)WHGDWPLPTKLPLVGGHEGS(A)GVVVGMGENVK | 40 | 81 |
| **Ser-tRNA^Ala^** | P00331 | LPLVGGHEGS(A)GVVVGMGENVK | 61 | 81 |
| **Ser-tRNA^Ala^** | P00331 | ES(A)LDFFS(A)R | 304 | 311 |
| **Ser-tRNA^Ala^** | P00358 | TASGNIIPSSTGAS(A)K | 199 | 213 |
| **Ser-tRNA^Ala^** | P00358 | KVVITAPSSTS(A)PMFVMGVNEEK | 116 | 137 |
| **Ser-tRNA^Ala^** | P00358 | IVSNS(A)SCTTNCLS(A)PLS(A)K | 144 | 160 |
| **Ser-tRNA^Ala^** | P00358 | LTGMS(A)FR | 226 | 232 |
| **Ser-tRNA^Ala^** | P00359 | YS(A)GEVSHDDKHIIVDGK | 54 | 70 |
| **Ser-tRNA^Ala^** | P00359 | IS(A)LSRPNVEVVS(A)LNDPFITNDYS(A)S(A)YMFK | 19 | 46 |
| **Ser-tRNA^Ala^** | P00359 | KVVITAPSSTS(A)PMFVMGVNEEK | 116 | 143 |
| **Ser-tRNA^Ala^** | P00359 | IVSNS(A)SCTTNCLS(A)PLS(A)K | 144 | 160 |
| **Ser-tRNA^Ala^** | P00359 | VINDS(A)FGIEEGLMTTVHSLTS(A)TQK | 161 | 184 |
| **Ser-tRNA^Ala^** | P00359 | TS(A)SGNIIPSSTGS(A)S(A)K | 199 | 213 |
| **Ser-tRNA^Ala^** | P00359 | LTGMS(A)FR | 226 | 232 |
| **Ser-tRNA^Ala^** | P00360 | IVSNS(A)SCTTNCLS(A)PLS(A)K | 144 | 160 |
| **Ser-tRNA^Ala^** | P00360 | TS(A)SGNIIPSSTGS(A)S(A)K | 199 | 213 |
| **Ser-tRNA^Ala^** | P00360 | LTGMS(A)FR | 226 | 232 |
| **Ser-tRNA^Ala^** | P00560 | S(A)HSSMVGFDLPQR | 170 | 182 |
| **Ser-tRNA^Ala^** | P00812 | HGLQTSIEDLGWSTELEPSMDES(A)QFVGK | 41 | 68 |
| **Ser-tRNA^Ala^** | P00812 | S(A)VHPETNGEGPIMCSYDVDGVDPLYIPATGTPVR | 240 | 273 |
| **Ser-tRNA^Ala^** | P02994 | VETGVIKPGMVVTFS(A)PAGVTTEVK | 265 | 288 |
| **Ser-tRNA^Ala^** | P02994 | FVPSKPMCVES(A)FSEYPPLGR | 402 | 421 |
| **Ser-tRNA^Ala^** | P05373 | SVILFGVPLIPGTKDPVGTAADDPS(A)GPVIQGIK | 85 | 117 |
| **Ser-tRNA^Ala^** | P05373 | MHTS(A)EFLETEPTEISSVLAGGYNHPLLR | 1 | 28 |
| **Ser-tRNA^Ala^** | P06168 | DLTHVEPPKDLDVILVS(A)PK | 179 | 197 |
| **Ser-tRNA^Ala^** | P06168 | GCLMGGIHGMFLS(A)QYDVLR | 261 | 279 |
| **Ser-tRNA^Ala^** | P06168 | GINSSYS(A)VWNDVTGK | 212 | 226 |
| **Ser-tRNA^Ala^** | P06168 | NDTFS(A)LIGYGSQGYGQGLNLR | 77 | 97 |
| **Ser-tRNA^Ala^** | P06168 | NLFTVEDS(A)IKR | 127 | 137 |
| **Ser-tRNA^Ala^** | P06168 | NS(A)LKPVFQDLYESTK | 333 | 347 |
| **Ser-tRNA^Ala^** | P06168 | S(A)S(A)IEDGWVPGK | 116 | 126 |
| **Ser-tRNA^Ala^** | P06168 | YGMDYMYDS(A)CSTTAR | 307 | 321 |
| **Ser-tRNA^Ala^** | P06168 | S(A)S(A)IEDGWVPGKNLFTVEDS(A)IK | 116 | 136 |
| **Ser-tRNA^Ala^** | P06168 | GSYVMNLLSDS(A)S(A)QSETWPS(A)IKPLLTK | 138 | 163 |
| **Ser-tRNA^Ala^** | P06168 | GSYVMNLLSDS(A)S(A)QSETWPAIKPLLTK | 138 | 163 |
| **Ser-tRNA^Ala^** | P06168 | S(A)QS(A)LS(A)VS(A)IGSGYVYQTTFER | 231 | 250 |
| **Ser-tRNA^Ala^** | P06168 | ENGHSPSES(A)FNETVEES(A)TQSLYPLIGK | 280 | 306 |
| **Ser-tRNA^Ala^** | P06168 | GS(A)LDWYPIFK | 323 | 332 |
| **Ser-tRNA^Ala^** | P06169 | MSS(A)NISETTS(A)MITDIS(A)TS(A)PS(A)EIDR | 128 | 151 |
| **Ser-tRNA^Ala^** | P07246 | YSGVCHTDLHS(A)WHGDWPLPVK | 67 | 87 |
| **Ser-tRNA^Ala^** | P07257 | YDYS(A)VAEQCPVK | 129 | 140 |
| **Ser-tRNA^Ala^** | P07257 | FVDESLLSTLPS(A)GK | 200 | 213 |
| **Ser-tRNA^Ala^** | P07703 | ES(A)NFDLINIDTSIANAFR | 51 | 68 |
| **Ser-tRNA^Ala^** | P10591 | AVGIDLGTTYSCVAHFS(A)NDRVDIIANDQGNR | 4 | 34 |
| **Ser-tRNA^Ala^** | P10592 | NQS(A)S(A)MNPS(A)NTVFDS(A)K | 55 | 69 |
| **Ser-tRNA^Ala^** | P15646 | IIS(A)LNSHMFLK | 252 | 262 |
| **Ser-tRNA^Ala^** | P15646 | MLIGMVDCVFS(A)DVAQPDQAR | 232 | 251 |
| **Ser-tRNA^Ala^** | P15646 | NMS(A)PGESVYGEKR | 110 | 122 |
| **Ser-tRNA^Ala^** | P15646 | LS(A)S(A)GIMGGLDELFIS(A)PGK | 151 | 168 |
| **Ser-tRNA^Ala^** | P15646 | S(A)NCIDSTVDS(A)ETVFS(A)R | 273 | 288 |
| **Ser-tRNA^Ala^** | P17076 | YGLNHVVS(A)LIENK | 134 | 146 |
| **Ser-tRNA^Ala^** | P22202 | AVGIDLGTTYSCVAHFS(A)NDR | 4 | 23 |
| **Ser-tRNA^Ala^** | P28834 | GQNVANPTS(A)MILSSTLMLNHLGLNEYATR | 293 | 321 |
| **Ser-tRNA^Ala^** | P38113 | DIVGS(A)IIKS(A)TNGGSHGVINVSVSES(A)S(A)IES(A)STR | 230 | 261 |
| **Ser-tRNA^Ala^** | P38113 | S(A)TNGGSHGVINVSVSEAAIEASTR | 238 | 261 |
| **Ser-tRNA^Ala^** | P38113 | ES(A)LDFFS(A)R | 307 | 314 |
| **Ser-tRNA^Ala^** | P38115 | IPALGLGTS(A)NPHEK | 33 | 46 |
| **Ser-tRNA^Ala^** | P38115 | ISSSIEFS(A)SLTKDELQELNDFGEKYPVR | 292 | 319 |
| **Ser-tRNA^Ala^** | P38891 | EIGWNNEDIHVPLLPGEQCGS(A)LTK | 343 | 366 |
| **Ser-tRNA^Ala^** | P38891 | LGS(A)NYS(A)PCILPQLQS(A)S(A)K | 220 | 236 |
| **Ser-tRNA^Ala^** | P38891 | ELVTS(A)PLDGTILEGVTR | 273 | 289 |
| **Ser-tRNA^Ala^** | P38891 | S(A)KQGELLES(A)FGSGTS(A)S(A)VVSPIK | 321 | 342 |
| **Ser-tRNA^Ala^** | P38891 | QVS(A)QWIS(A)DIQYGR | 367 | 379 |
| **Ser-tRNA^Ala^** | P40075 | AVS(A)DVWSDLEAEFK | 100 | 113 |
| **Ser-tRNA^Ala^** | P47176 | ELVTS(A)PLDGTILEGVTR | 256 | 272 |
| **Ser-tRNA^Ala^** | Q04894 | MGS(A)DHYIATLEEGDWGEK | 222 | 239 |

**S3 Table –** **B: Peptides with Gly→Ser misincorporation detected by LC-MS/MS.**

| **Strain** | **Uniprot ID** | **Peptide** | **Start** | **End** |
| --- | --- | --- | --- | --- |
| **Control** | P00330 | SANLMAS(G)HWVAISS(G)AAGGLGSLAVQYAK | 165 | 192 |
| **Control** | P00330 | SANLMAGHWVAISS(G)AAGGLGSLAVQYAK | 165 | 192 |
| **Control** | P00330 | ATDS(G)S(G)AHS(G)VINVSVSEAAIEASTR | 235 | 258 |
| **Control** | P00330 | ATDS(G)S(G)AHGVINVSVSEAAIEASTR | 235 | 258 |
| **Control** | P00330 | ANS(G)TTVLVS(G)MPAS(G)AKCCSDVFNQVVK | 262 | 287 |
| **Control** | P00331 | SANLRAS(G)HWAAISS(G)AAGGLGSLAVQYAK | 165 | 192 |
| **Control** | P00331 | EKDIVSAVVKATNS(G)S(G)AHS(G)IINVSVSEAAIEASTR | 225 | 258 |
| **Control** | P00359 | VINDAFS(G)IEES(G)LMTTVHSLTATQKTVDGPSHK | 161 | 192 |
| **Control** | P00812 | AVHPETNS(G)ES(G)PIMCSYDVDS(G)VDPLYIPATS(G)TPVR | 240 | 273 |
| **Control** | P02994 | NMITS(G)TSQADCAILIIAS(G)GVGEFEAGISK | 101 | 129 |
| **Control** | P02994 | VETS(G)VIKPS(G)MVVTFAPAS(G)VTTEVK | 265 | 288 |
| **Control** | P06168 | S(G)LKQINFGGTVETVYER | 47 | 63 |
| **Control** | P06168 | S(G)CLMS(G)S(G)IHS(G)MFLAQYDVLR | 261 | 279 |
| **Control** | P07246 | VS(G)DLAGIK | 112 | 119 |
| **Control** | P0C2J3 | IINLS(G)ER | 1469 | 1475 |
| **Control** | P16550 | ITEKPELINDILLECS(G)FPNTSS(G)QKPNEYNY | 292 | 321 |
| **Control** | P16861 | QAAS(G)NLISQS(G)IDALVVCGGDGSLTGADLFR | 290 | 319 |
| **Control** | P42950 | TPAHNTNIQYTYNINDNFQSAS(G)SIPR | 106 | 131 |
| **Control** | Q04439 | VIGLS(G)QEEQDQIFR | 282 | 295 |
| **Control** | Q12166 | YAVENAPDDVS(G)IQCLVQSR | 115 | 133 |
| **Ser-tRNA^Gly^** | P00330 | YSS(G)VCHTDLHAWHS(G)DWPLPVKLPLVGGHEGAGVVVGMGENVK | 40 | 81 |
| **Ser-tRNA^Gly^** | P00330 | SANLMAS(G)HWVAISGAAGGLS(G)SLAVQYAKAMS(G)YR | 165 | 197 |
| **Ser-tRNA^Gly^** | P00330 | SANLMAS(G)HWVAISGAAGGLGSLAVQYAKAMGYR | 165 | 192 |
| **Ser-tRNA^Gly^** | P00330 | SANLMAGHWVAISS(G)AAS(G)S(G)LGSLAVQYAKAMGYR | 165 | 192 |
| **Ser-tRNA^Gly^** | P00330 | ATDS(G)S(G)AHS(G)VINVSVSEAAIEASTR | 235 | 258 |
| **Ser-tRNA^Gly^** | P00330 | ANS(G)TTVLVS(G)MPAS(G)AKCCSDVFNQVVK | 262 | 287 |
| **Ser-tRNA^Gly^** | P00331 | SANLRAS(G)HWAAISS(G)AAS(G)S(G)LS(G)SLAVQYAK | 165 | 192 |
| **Ser-tRNA^Gly^** | P00358 | VVITAPSSTAPMFVMS(G)VNEEKYTSDLKIVSNASCTTNCLAPLAK | 117 | 160 |
| **Ser-tRNA^Gly^** | P00359 | DPANLPWS(G)SSNVDIAIDSTS(G)VFKELDTAQK | 79 | 108 |
| **Ser-tRNA^Gly^** | P00359 | VINDAFS(G)IEES(G)LMTTVHSLTATQK | 161 | 184 |
| **Ser-tRNA^Gly^** | P00359 | VVITAPSSTAPMFVMS(G)VNEEKYTSDLKIVSNASCTTNCLAPLAK | 117 | 160 |
| **Ser-tRNA^Gly^** | P00359 | VINDAFS(G)IEES(G)LMTTVHSLTATQKTVDS(G)PSHK | 161 | 192 |
| **Ser-tRNA^Gly^** | P00360 | LISWYDNEYS(G)YSAR | 308 | 321 |
| **Ser-tRNA^Gly^** | P00812 | AVHPETNS(G)EGPIMCSYDVDS(G)VDPLYIPATS(G)TPVR | 240 | 273 |
| **Ser-tRNA^Gly^** | P02994 | NMITS(G)TSQADCAILIIAS(G)GVGEFEAGISK | 101 | 129 |
| **Ser-tRNA^Gly^** | P06168 | S(G)INSSYAVWNDVTGK | 212 | 226 |
| **Ser-tRNA^Gly^** | P06168 | LLDYFKNDTFALIS(G)YS(G)SQGYGQGLNLR | 71 | 97 |
| **Ser-tRNA^Gly^** | P06168 | S(G)SYVMNLLSDAAQSETWPAIKPLLTK | 138 | 163 |
| **Ser-tRNA^Gly^** | P06168 | S(G)CLMS(G)S(G)IHS(G)MFLAQYDVLR | 261 | 279 |
| **Ser-tRNA^Gly^** | P06168 | ENS(G)HSPSEAFNETVEEATQSLYPLIGK | 280 | 306 |
| **Ser-tRNA^Gly^** | P07246 | VS(G)DLAGIK | 112 | 119 |
| **Ser-tRNA^Gly^** | P07257 | S(G)LS(G)NPLLYDS(G)VER | 154 | 166 |
| **Ser-tRNA^Gly^** | P15646 | ISVEEPSKEDS(G)VPPTKVEYR | 123 | 142 |
| **Ser-tRNA^Gly^** | P25294 | LYDLLS(G)VSPSANEQELKK | 7 | 24 |
| **Ser-tRNA^Gly^** | P28241 | VVTNPSAYTDAVSVCPNLYS(G)DILSDLNSS(G)LSAS(G)SLS(G)LTPSANIS(G)HK | 243 | 282 |
| **Ser-tRNA^Gly^** | P28241 | ANPTALLLSSVMMLNHMS(G)LTNHADQIQNAVLSTIASGPENR | 308 | 348 |
| **Ser-tRNA^Gly^** | P28834 | S(G)LWHTPADQTGHS(G)SLNVALR | 90 | 109 |
| **Ser-tRNA^Gly^** | P32324 | KIWCFS(G)PDS(G)NS(G)PNLVIDQTK | 632 | 651 |
| **Ser-tRNA^Gly^** | P32611 | S(G)NCLPISMIPIS(G)TIIHNVGITPVGPGK | 238 | 264 |
| **Ser-tRNA^Gly^** | P38891 | EIS(G)WNNEDIHVPLLPGEQCGALTK | 343 | 366 |
| **Ser-tRNA^Gly^** | P40093 | DNAISIPS(G)IISS(G)MNPNWNEDTSDESFDR | 160 | 187 |
| **Ser-tRNA^Gly^** | P53111 | VS(G)S(G)EFYNYCS(G)PFIDVR | 232 | 247 |
| **Ser-tRNA^Gly^** | Q04439 | VIGLS(G)QEEQDQIFR | 282 | 295 |
| **Ser-tRNA^Gly^** | Q12166 | YAVENAPDDVS(G)IQCLVQSR | 115 | 133 |
| **Ser-tRNA^Gly^** | Q99344 | MDCKILVLGAGGLS(G)CEILK | 1 | 19 |
