# Supplementary material for: Errors in protein synthesis increase the level of saturated fatty acids and affect the overall lipid profiles of yeast
Source: PLoS One. 2018 Aug 27;13(8):e0202402. doi: 10.1371/journal.pone.0202402 (PMC6110467; doi:10.1371/journal.pone.0202402)
Supplement: S1 Protocol — (PDF) [file pone.0202402.s014.pdf]

S1 Protocol. Determination of β-galactosidase activity and quantity.

β-galactosidase assay

S. cerevisiae strains co-expressing pGL-C1 and either pRS315 (control strain), pUA268 or pUA269 were collected in exponential phase, washed and resuspended in PBS 1x. After protein isolation (see above), 2.5 µL crude extracts were mixed in 500 µL of Z-buffer (60 mM Na_2HPO_4, 40 mM $NaH_2PO.2H_2O$, 10 mM KCl, 1 mM $MgSO_4.7H_2O$, 50 mM 2-mercaptoethanol, pH 7). For thermal inactivation, the mixes were incubated at 52 ^oC for 10 min to promote β-gal unfolding. Samples were then kept on ice for 30 min for protein refolding, after which followed the measurement of β-gal activity. Mixes were incubated at 37 ^oC, and the reaction was initiated by adding 100 µL of ONPG (4 mg/mL). After 10 min, the reaction was terminated by adding 250 µL of 1 M Na_2CO_3 and vigorously vortexed. The optical density was measured at 420 nm and the activity calculated according to the following equation: Act=OD_{420} x 0.85 / (0.0045 x total protein x extract volume x time). OD_{420} is the optical density of the product, o-nitrophenol, at 420 nm; the factor 0.85 corrects for the reaction volume; the factor 0.0045 is the optical density of 1 nmol/mL solution of o-nitrophenol; protein concentration is expressed in mg/ml; extract volume is the volume assayed in mL; time is in minutes; specific activity is expressed as nmol/min/mg protein.

Anti β-gal western blot

S. cerevisiae strains co-expressing pGL-C1 and either pRS315 (control strain), pUA268 or pUA269 were collected in exponential phase. Protein isolation was performed as described above. Protein extracts (50 µg) were denatured for 5 min at 95^oC and separated in 10% resolving and 4% stacking acrylamide gels. Proteins were blotted onto a hydrated nitrocellulose membrane (Hybond ECL, Amersham) in a Bio-Rad wet transfer system, for 1 h at 4 ^oC. Afterwards, membranes were stained with Ponceau, de-stained and blocked for 1h at RT in TBS-T and 5% BSA. Membranes were then incubated

overnight at 4 ºC with the primary antibody mix comprising the anti-β-gal (1:5000, Invitrogen) and the anti-ADH (1:1000, Rockland) in TBS-T and 1% BSA. Membranes were washed in TBS-T and incubated with the secondary antibody (anti-rabbit, 1:10000, IFRDye680 LI-COR antibody, Odyssey) for 1 h at RT. Detection was achieved with an Odyssey infrared imaging system (LI-COR Biosciences) and images obtained with the Odyssey v3 software.
