# Supplementary material for: Errors in protein synthesis increase the level of saturated fatty acids and affect the overall lipid profiles of yeast
Source: PLoS One. 2018 Aug 27;13(8):e0202402. doi: 10.1371/journal.pone.0202402 (PMC6110467; doi:10.1371/journal.pone.0202402)
Supplement: S2 Protocol — (PDF) [file pone.0202402.s015.pdf]

S2 Protocol. Detection of tRNA by northern blot.

25 µg of RNA were resolved in a 15% polyacrylamide gel, containing 8 M urea, at 500 V for 16 h. After staining and RNA identification, the section of the gels containing the tRNAs was transferred to a nitrocellulose membrane (Hybond N, Amersham) with a Semi-Dry Trans Blotting system (Bio-Rad). Nitrocellulose membranes obtained after the polyacrylamide gel electrophoresis were subjected to a Northern blot. Probes were prepared by phosphorylating 10 pmol of dephosphorylated oligonucleotide, 5’-TTAACCGCTCGGACAAGTT, with 3 µl of ɣ-^{32}P-ATP (5000 Ci/mmol) (Perkin Elmer) in 1x T4 Kinase buffer, 2 µl of 0.1 mM spermidine and 1.5 µl of T4 Kinase (Takara). This reaction was carried out by incubating the reactions for 1 h at 37 ^{o}C. Probes were purified with 100 µl phenol/chloroform/isoamyl alcohol (25:24:1). Membrane and probes were incubated in hybridization solution (6.6x SSPE, 5x Denhardt’s solution, 1% SDS), overnight, at a temperature 5 ^{o}C lower than the probe melting temperature (Tm). Hybond-N membranes were washed with washing solution (2x SSPE, 0.5% SDS), wrapped in a plastic bag and exposed for 24 h to a K-screen and scanned using Molecular Imager FX (Bio-Rad).
